# Hypoellipticity of analytic differential operators in general ultradifferentiable classes

**DOI:** 10.1007/s11868-026-00803-0

**Published:** 2026-09-04

**Authors:** Stefan Fürdös

**Affiliations:** Institute of Mathematics, University of Vienna, Oskar-Morgenstern-Platz 1, 1090 Vienna, Austria

**Keywords:** Ultradifferentiable hypoellipticity, Ultradifferentiable wavefront set, Analytic pseudodifferential operators, (non-)quasianalyticity, Primary 35B65, Secondary 35H10, 35A18, 26E10

## Abstract

We show that analytic pseudodifferential and Fourier integral operators behave well for ultradifferentiable classes satisfying minimal regularity properties. As an application we investigate the ultradifferentiable regularity properties of several examples of analytic differential operators. In particular we extend Treves’ characterization of the hypoellipticity of analytic operators of principal type to the ultradifferentiable category.

## Introduction

The focus of this paper is the study of the ultradifferentiable regularity properties of analytic differential operators with respect to a large family of ultradifferentiable classes. In recent years there has been an increased interest in the study of the regularity of partial differential equations with respect to ultradifferentiable classes, see e.g. [1, 2, 10, 18, 19] or [20].

We take a slightly different approach compared with the predominant point of view of the literature by asking if the regularity properties of a given analytic partial differential operator *P* with respect to some ultradifferentiable class influence the regularity properties of *P* regarding a different ultradifferentiable class. For example, by the definition of hypoellipticity, if a differential operator is hypoelliptic with respect to some ultradifferentiable class then this knowledge does not in general give information whether the operator is hypoelliptic in terms of a different class. But several results, e.g. [8, 13, 29] or [12], see also [35], indicate that there is a connection between nondegeneracy conditions on the operators and the property that hypoellipticity with respect to one ultradifferentiable class implies hypoellipticity for any larger class.

In fact, our first main statement, Theorem 3.14, shows how operators acting on distributions transforms the wavefront set with respect to suitable general ultradifferentiable classes in terms of the ultradifferentiable wavefront set of their distributional kernel by generalizing a result of Hörmander [22] in the case of Denjoy-Carleman classes given by weight sequences to a wide family of ultradifferentiable classes which includes, among others, ultradifferentiable classes in the sense of Braun–Meise–Taylor [9]. We might see this also as a continuation of the work done in [16] on the study of microlocal analysis in general ultradifferentiable classes. In particular, our statement implies that an analytic parametrix is not only a smooth parametrix but also an ultradifferentiable parametrix. Another direct consequence is that analytic pseudodifferential operators are microlocal with respect to suitable ultradifferentiable classes and analytic Fourier Integral Operators transform the ultradifferentiable wavefront set as expected, cf. Sect. 4.

We may refer to ultradifferentiable classes, for which our results hold, as semiregular. For a precise definition of the classes we consider, see the next section and the beginning of Sect. 3. The results mentioned above induce directly that elliptic analytic pseudodifferential operators are hypoelliptic with respect to any semiregular ultradifferentiable class. This generalizes well-known results on the ultradifferentiable regularity of elliptic differential operators with analytic coefficients by [22] in the case of Denjoy-Carleman classes, for other classes see [1] and [16]. But obviously we can also use our methods to prove that other differential operators, which possess an analytic parametrix, are actually ultradifferentiable hypoelliptic. In particular, we are able to extend Treves’ characterization of hypoellipticity of analytic differential operators of principal type to the ultradifferentiable setting, which can formulated slightly loosely in the following way:

### Theorem

Let *P* be an analytic differential operator of principal type and $\mathcal {U}$ be a semiregular ultradifferentiable class. Then *P* is smooth hypoelliptic if and only if *P* is hypoelliptic with respect to $\mathcal {U}$.

For a precise formulation see Theorem 2.8 and Theorem 5.2. Other operators we are looking at are those considered in [13] and in [30].

Therefore our results, summarized in the next section, show that operators with very nice nondegeneracy properties have nice regularity properties for a wide family of ultradifferentiable classes. Using more general ultradifferentiable classes in the study of regularity of PDOs might give more precise information than the usual approach with Gevrey classes. In the rest of the introduction we try to give an overview of the existing literature in order to indicate how the results of this paper fit into it. We may start by noticing that analytic hypoellipticity of operators does not generally imply hypoellipticity in the smooth category: For example

$$\begin{aligned} P=\left( D_1+ix_1^{2\ell }D_2\right) ^2+cD_2, \end{aligned}$$

where $\ell \in \mathbb {N}$ and $c\in \mathbb {C}\!\setminus \!\{0\}$, is analytic hypoelliptic but not hypoelliptic, cf. [32] and for other examples in this regard see [35]. In fact, [32] showed that *P* is $\mathcal {G}^s$-hypoelliptic for $1\le s\le 4\ell /(2\ell -1)$ where $\mathcal {G}^s$ denotes the Gevrey class of degree s≥1, i.e. the ultradifferentiable class associated to the weight sequence $k!^s$, cf. Definition 2.1. In contrast, it is a classical statement that the heat operator $\partial _t-\Delta _x$ is smooth and $\mathcal {G}^s$-hypoelliptic if and only if s≥2. More generally the characterization of hypoellipticity of differential operators with constant coefficients [21], see also [24] and [35], implies that this is generally true for hypoelliptic operators *P* with constant coefficients: If *P* is smooth hypoelliptic then there is some $s_0\ge 1$ such that *P* is $\mathcal {G}^s$-hypoelliptic for $s\ge s_0$, where $s_0$ depends on the geometry of the symbol P(ξ) of *P*. This indicates that there might be a connection between the geometry of the symbol of some operator with its hypoelliptic properties with respect of Gevrey or, more generally, ultradifferentiable classes.

Historically, beginning with the study of differential operators with constant operators, the Gevrey classes $\mathcal {G}^s$ have been used to interpolate between the algebra of smooth functions $\mathcal {C}^\infty$ and the space of real-analytic functions $\mathcal {C}^\omega$, see [35] and the references therein. A current example of this approach is the ongoing investigation of the problem of analytic hypoellipticity of sum of squares of analytic real vector fields which satisfy the Hörmander condition, see for example [3, 6, 7, 11]. The main reason, why this approach is sensible is that if *P* is an analytic sum of squares operator satisfying the condition of Hörmander then there is some s≥1 such that *P* is $\mathcal {G}^s$-hypoelliptic, cf. [14] and if *P* is $\mathcal {G}^s$-hypoelliptic then *P* is $\mathcal {G}^t$-hypoelliptic for any $t\ge s\ge 1$. The last statement is actually a corollary of Métivier’s inequality, which in turn characterizes analytic and Gevrey hypoellipticity for operators which are $L^2$-solvable, see [29] and [8].

With respect to ultradifferentiable classes Métivier’s inequality has been generalized by Cordaro and the author [12] to Denjoy-Carleman classes $\mathcal {E}^{\{\textbf{M}\}}$ given by admissible weight sequences $\textbf{M}$. Here admissible weight sequences are semiregular (see Definition 2.3) and satisfy the following condition

**Figure:**
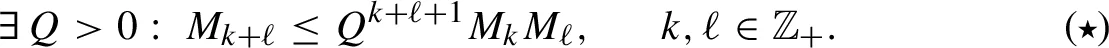

The important point here is that we do not require in [12] that $\mathcal {E}^{\{ \textbf{M} \}} ( \Omega )$ is non-quasianalytic. Recall that a subalgebra *E* of $\mathcal {C}^\infty (\Omega )$ is non-quasianalytic if there are non-trivial ultradifferentiable functions with compact support, i.e. $E\cap \mathcal {C}^\infty _0(\Omega )\ne \{0\}$. The well-known Denjoy-Carleman Theorem (see e.g. [23]) states that $\mathcal {E}^{\{ \textbf{M} \}} ( \Omega )$ is non-quasianalytic if and only if

**Figure:**
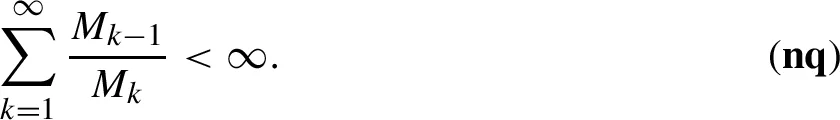

We say that the weight sequence $\textbf{M}$ is quasianalytic if ($\textbf{nq}$) is not satisfied.

As in the Gevrey setting it is a direct consequence of the ultradifferentiable Métivier inequality that $\{\textbf{M}\}$-hypoellipticity, $\textbf{M}$ being admissible, of an $L^2$-solvable differential operator implies $\{\textbf{N}\}$-hypoellipticity for any larger class $\mathcal {E}^{\{ \textbf{N} \}} ( \Omega )$, where $\textbf{N}$ is admissible, and smooth hypoellipticity. But we need to point out that admissibility is a rather strict condition for weight sequences. More precisely, if $\textbf{M}$ satisfies (⋆) then there is some s>1 such that $\mathcal {E}^{\{\textbf{M}\}}\subseteq \mathcal {G}^s$, cf. [28]. This means that many reasonable Denjoy-Carleman classes are not admissible: In particular the results in [12] do not cover the classes given by the weight sequences $\textbf{N}^q=q^{k^2}$, q>1, which are semiregular (cf. Definition 2.3). Another interesting example of a semiregular weight sequence, which is not admissible, is given in [16]: There exists a semiregular weight sequence $\textbf{L}$ with the following properties:

- $$\textbf{L}$$ is quasianalytic.
- $$\mathcal {E}^{\{\textbf{M}\}}\nsubseteq \mathcal {G}^s$$ for all s>1.

Hence using the ultradifferentiable Métivier inequality we could not infer that an analytic hypoelliptic differential operator of principal type is hypoelliptic with respect to the classes given by either the sequence $\textbf{N}$ or $\textbf{L}$, which is true due our main Theorem, cf. Theorem 2.8. But in fact the setting we work in this paper is still much larger regarding the scope of ultradifferentiable classes we consider: First, in [12] we only worked with Roumieu classes given by weight sequences, but here we consider also Beurling classes (see Definition 2.1 for the difference).

Moreover, as alluded to above, the setting we work with in this paper includes ultradifferentiable classes given by weight functions in the sense of Braun–Meise–Taylor [9]. Gevrey classes can be realized both as Denjoy-Carleman classes and as Braun-Meise-Taylor classes, but in general the classes in the sense of Braun–Meise–Taylor classes cannot be described as Denjoy-Carleman classes and vice-versa, cf. [4]. In fact, we work in the setting of ultradifferentiable classes given by weight matrices, i.e. families of weight sequences, introduced by [34]. These classes include both Denjoy-Carleman classes and Braun-Meise-Taylor classes and using weight matrices allows to take an unified approach. We may remark that the advantage to work in the setting of weight matrices is particularly apparent in the study of the Problem of Iterates, see [17]. Here we note that we can construct semiregular quasianalytic classes given by weight matrices, which are not contained in any semiregular Denjoy-Carleman classes, cf. Corollary B.2.

The paper is organized in the following way: In Section 2 the results on the ultradifferentiable hypoellipticity are summarized for easier reference in terms of Denjoy-Carleman and Braun-Meise-Taylor classes. In Section 3 we first recall the basic facts about the ultradifferentiable wavefront set associated to classes given by weight matrices from [16] and continue to prove the generalization of Hörmander’s theorem on the ultradifferentiable microlocal transform properties of operators in terms of their distributional kernel.

In Sect. 4 we apply this theorem to analytic pseudodifferential operators and analytic Fourier integral operators. In particular we prove the microlocal regularity theorem for classical analytic pseudodifferential operators in the category of semiregular ultradifferentiable classes. Section 5 is devoted to the generalization of Treves’ Theorem on hypoelliptic differential operators of principal type. Finally, in Sect.6 we prove the ultradifferentiable version of the main result of [30].

We have included two appendices, which might be of interest. In the first appendix we quickly summarize the basic theory on ultradifferentiable hypoellipticity of differential operators with constant coefficients in terms of classes given by weight matrices. This might be well-known to experts in the field, but it seems not to have been written down in the literature beyond the Gevrey case.

The second appendix is basically devoted to show that there is a quasianalytic semiregular ultradifferentiable class given by a weight matrix (even a weight function) which is *not* contained in any semiregular Denjoy-Carleman class.

## Statement of main results

### Definition 2.1

A sequence $\textbf{M}=(M_k)_k$ of positive numbers is called a weight sequence if $M_0=1$, $\root k \of {M_k}\rightarrow \infty$ and

2.1 $$\begin{aligned} M_k^2\le M_{k-1}M_{k+1},\qquad k\in \mathbb {N}. \end{aligned}$$

The Roumieu class $\mathcal {E}^{\{ \textbf{M} \}} ( \Omega )$ associated to $\textbf{M}$ on an open set $\Omega \subseteq \mathbb {R}^n$ consists of the smooth functions $f\in \mathcal {C}^\infty (\Omega )$ such that for each compact set $K\subseteq \Omega$ there are constants C,h>0 so that the following estimate holds:

2.2 $$\begin{aligned} \sup _{x\in K}\,|D^\alpha f(x)|\le Ch^{|\alpha |}M_{|\alpha |},\qquad \;\,\forall \,\alpha \in \mathbb {Z}_+^n. \end{aligned}$$

On the other hand a smooth function $f\in \mathcal {C}^\infty (\Omega )$ is an element of the Beurling class $\mathcal {E}^{( \textbf{M} )} ( \Omega )$ associated to $\textbf{M}$ if for all compact sets $K\subseteq \Omega$ and every h>0 there is a constant C>0 such that (2.2) is satisfied.

### Definition 2.2

A weight functions ω is a non-decreasing continuous function $\omega : [0,\infty )\rightarrow [0,\infty )$ such that ω(t)=0 for t∈[0,1] and the following conditions hold:

**Figure:**
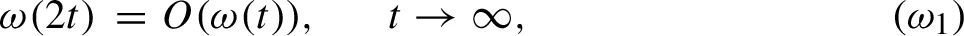

**Figure:**
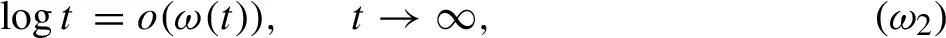

**Figure:**
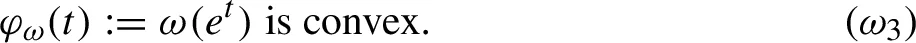

We need also the conjugate function of $\varphi _\omega$:

$$\begin{aligned} \varphi ^*_\omega (t)=\sup _{s\ge 0} (st-\varphi _\omega (s)). \end{aligned}$$

The Roumieu class $\mathcal {E}^{\{ \omega \}} ( \Omega )$ associated to ω is the space of functions $f\in \mathcal {C}^\infty (\Omega )$ such that for every compact set $K\subseteq \Omega$ there are constants C,h>0 so that the following estimate is satisfied

2.3 $$\begin{aligned} \sup _{x\in K}|D^\alpha f(x)|\le Ce^{\varphi ^*_\omega (h{|\alpha |})},\quad \alpha \in \mathbb {Z}_+^n. \end{aligned}$$

A function $f\in \mathcal {C}^\infty (\Omega )$ is an element of the Beurling class $\mathcal {E}^{( \omega )} ( \Omega )$ if for each compact set $K\subseteq \Omega$ and every h>0 there is a constant C>0 such that (2.3) holds.

We will use the notation $[*]=\{*\},(*)$ ($*=\textbf{M},\omega$ etc.) if the concerned statements hold for Roumieu and Beurling classes alike.

### Definition 2.3

1. A weight sequence $\textbf{M}$ is semiregular if the following conditions hold:
  2.4 $$\begin{aligned} \lim _{k\rightarrow \infty }\frac{\root k \of {M_k}}{k}=\infty , \end{aligned}$$
  2.5 $$\begin{aligned} \;\,\exists \,Q>0:\quad M_{k+1}\le Q^{k+1}M_k, \qquad \;\,\forall \,k\in \mathbb {Z}_+. \end{aligned}$$
2. We say that a weight function is semiregular if

2.6 $$\begin{aligned} \omega (t)=o(t),\qquad t\rightarrow \infty . \end{aligned}$$

### Remark 2.4

If $\textbf{M}$ (resp. ω) is semiregular then $\mathcal {E}^{[ \textbf{M} ]} ( \Omega )$ (resp. $\mathcal {E}^{[ \omega ]} ( \Omega )$) is closed under derivation and contains strictly the class $\mathcal {C}^\omega (\Omega )$ of analytic functions: In the case of weight sequences condition (2.4) gives instantly that $\mathcal {C}^\omega (\Omega )=\mathcal {G}^1(\Omega )\subsetneq \mathcal {E}^{[ \textbf{M} ]} ( \Omega )$. Moreover, (2.5) implies that if $f\in \mathcal {E}^{[ \textbf{M} ]} ( \Omega )$ then $D^\alpha f\in \mathcal {E}^{[ \textbf{M} ]} ( \Omega )$ for any $\alpha \in \mathbb {Z}_+^n$.

Regarding classes given by weight functions, derivation closedness of $\mathcal {E}^{[ \omega ]} ( \Omega )$ follows from the convexity of $\varphi _\omega$ , cf. [9]. On the other hand, (2.6) gives that $\mathcal {C}^\omega (\Omega )\subsetneq \mathcal {E}^{[ \omega ]} ( \Omega )$, see [34].

### Definition 2.5

Let *P* be a pseudodifferential operator in $\Omega \subseteq \mathbb {R}^n$.

1. If $\textbf{M}$ is a weight sequence then we say that *P* is $[\textbf{M}]$-hypoelliptic if
  $$\begin{aligned} {{\,\mathrm{sing\, supp}\,}}_{[\textbf{M}]} Pu={{\,\mathrm{sing\, supp}\,}}_{[\textbf{M}]} u \end{aligned}$$
  for all $$u\in \mathcal {E}^\prime (\Omega )$$.
2. If ω is a weight function then *P* is [ω]-hypoelliptic if
  $$\begin{aligned} {{\,\mathrm{sing\, supp}\,}}_{[\omega ]}Pu={{\,\mathrm{sing\, supp}\,}}_{[\omega ]} u \end{aligned}$$
  for all $$u\in \mathcal {E}^\prime (\Omega )$$.

Here ${{\,\mathrm{sing\, supp}\,}}_{[*]}u$ denotes the ultradifferentiable singular support of a distribution *u* which is defined in the obvious way. We can now state the main results of this article. The first one extends statements of [22] in the Roumieu case, for the Beurling case see [16], for analytic partial differential operators.

### Theorem 2.6

Let *A* be an elliptic analytic pseudodifferential operator in Ω. Then *A* is $[\textbf{M}]$-hypoelliptic for every semiregular weight sequence and [ω]-hypoelliptic for all semiregular weight functions

The simplest class of non-elliptic operators are the operators of principal type:

### Definition 2.7

A differential operator *P* of order *d* with principal symbol $p_d$ on Ω is of principal type^1^ if

$$\begin{aligned} |p_d(x,\xi )|+|d_\xi p_d(x,\xi )|\ne 0 \end{aligned}$$

for all $$(x,\xi )\in T^*\Omega \!\setminus \!\{0\}$$.

Treves [38] proved that a differential operator of principal type with analytic coefficients is hypoelliptic if and only if it is analytic hypoelliptic. We can extend this equivalence to include any semiregular ultradifferentiable class:

### Theorem 2.8

Let *A* be a differential operator of principal type with analytic coefficients, $\textbf{M}$ be a semiregular weight sequence and ω be a semiregular weight function. Then the following statements are equivalent:

1. *A* is smooth hypoelliptic.
2. *A* is $$\{\textbf{M}\}$$-hypoelliptic.
3. *A* is $$(\textbf{M})$$-hypoelliptic.
4. *A* is $$\{\omega \}$$-hypoelliptic.
5. *A* is (ω)-hypoelliptic.
6. *A* is analytic hypoelliptic.

Our methods also allow us to generalize a theorem of Métivier [30] on the analytic hypoellipticity of operators with multiple characteristics (for the analogous result regarding smooth hypoellipticity see [5]):

### Theorem 2.9

Let *P* be a classical analytic pseudodifferential operator of order *m* with symbol

$$\begin{aligned} p(x,\xi )\sim \sum _{j=0}^\infty p_{m-j}(x,\xi ) \end{aligned}$$

and suppose that ${{\,\textrm{Char}\,}}P$ is a symplectic analytic submanifold of $T^*\Omega \!\setminus \!\{0\}$. Assume further that there is some $k\in \mathbb {N}$ such that $p_m$ vanishes exactly of order *k* on ${{\,\textrm{Char}\,}}P$ and that $p_{m-j}$ vanishes of order k-2j on ${{\,\textrm{Char}\,}}P$ for j≤k/2. If finally *P* is *k*/2-subelliptic then *P* is $[\textbf{M}]$-hypoelliptic in Ω for any semiregular weight sequence $\textbf{M}$ and [ω]-hypoelliptic for all semiregular weight functions ω.

Finally our approach also leads directly to an extension of the main statements in [13]: Let $k\in \mathbb {N}$ and $\lambda \in \mathbb {C}$. We consider the linear differential operator

### Theorem 2.10

Let k≥2 be an even integer, $\textbf{M}$ be a semiregular weight sequence, ω a weight function with ω(t)=o(t), Ω be an open set with $\Omega \cap \{t=0\}\ne \emptyset$.

Then the following statements are equivalent:

1. $Q_\lambda$ is hypoelliptic in Ω.
2. $Q_\lambda$ is $\{\textbf{M}\}$-hypoelliptic in Ω.
3. $Q_\lambda$ is $(\textbf{M})$-hypoelliptic in Ω.
4. $Q_\lambda$ is $\{\omega \}$-hypoelliptic in Ω.
5. $Q_\lambda$ is (ω)-hypoelliptic in Ω.
6. $Q_\lambda$ is analytic hypoelliptic in Ω.
.

## Microlocal analysis in general ultradifferentiable classes

### Definition 3.1

A weight matrix $\mathfrak {M}$ is a collection of weight sequences such that for any pair $\textbf{M},\textbf{N}\in \mathfrak {M}$ we have either $M_j\le N_j$ for all $j\in \mathbb {Z}_+$ or $N_j\le M_j$ for all $j\in \mathbb {Z}_+$.

If $\Omega \subseteq \mathbb {R}^n$ is an open set then a smooth function $f\in \mathcal {C}^\infty \left( \Omega \right)$ is an element of the Roumieu class $\mathcal {E}^{\{ \mathfrak {M} \}} ( \Omega )$ associated to the weight matrix $\mathfrak {M}$ if for all compact sets $K\subseteq \Omega$ there are constants C,h>0 and some weight sequence $\textbf{M}\in \mathfrak {M}$ such that

3.1 $$\begin{aligned} \sup _{x\in K}|D^\alpha f(x)|\le Ch^{|\alpha |}M_{|\alpha |}\qquad \;\,\forall \,\alpha \in \mathbb {Z}_+^n. \end{aligned}$$

On the other hand *f* is in the Beurling class $\mathcal {E}^{( \mathfrak {M} )} ( \Omega )$ associated to $\mathfrak {M}$ if for every compact set $K\subseteq \Omega$, every $\textbf{M}\in \mathfrak {M}$ and all h>0 there is a constant C>0 such that (3.1) is satisfied.

### Definition 3.2

Let $\mathfrak {M}$ be a weight matrix and *P* be a linear differential operator with coefficients in $\mathcal {E}^{[ \mathfrak {M} ]} ( \Omega )$. Then we say that *P* is $[\mathfrak {M}]$-hypoelliptic at $x_0\in \Omega$ if there is a neighborhood $U_0$ of $x_0$ such that

$$\begin{aligned} {{\,\mathrm{sing\, supp}\,}}_{[\mathfrak {M}]}Pu={{\,\mathrm{sing\, supp}\,}}_{[\mathfrak {M}]} u \end{aligned}$$

for all $$u\in \mathcal {D}^\prime (U_0)$$.

The operator *P* is $[\mathfrak {M}]$-hypoelliptic in an open set $U\subseteq \Omega$ if *P* is $[\mathfrak {M}]$-hypoelliptic at every point *x* of *U*.

### Definition 3.3

Let $\mathfrak {M}$ be a weight matrix.

1. We say that $\mathfrak {M}$ is *R*-semiregular if
  3.2 $$\begin{aligned} \;\,\forall \,\textbf{M}\in \mathfrak {M}\;\,\lim _{j\rightarrow \infty }\left( \frac{M_j}{j!}\right) ^{1/j}=\infty \end{aligned}$$
  and
  3.3 $$\begin{aligned} \;\,\forall \,\textbf{M}\in \mathfrak {M}\,\;\,\exists \,\textbf{N}\in \mathfrak {M}\,\;\,\exists \,Q>0:\;\, M_{j+1}\le Q^{j+1}N_j\qquad \;\,\forall \,j\in \mathbb {Z}_+ \end{aligned}$$
  hold.
2. The weight matrix $\mathfrak {M}$ is *B*-semiregular if (3.2) and
  3.4 $$\begin{aligned} \;\,\forall \,\textbf{N}\in \mathfrak {M}\,\;\,\exists \,\textbf{M}\in \mathfrak {M}\,\;\,\exists \,Q>0:\;\, M_{j+1}\le Q^{j+1}N_j\qquad \;\,\forall \,j\in \mathbb {Z}_+ \end{aligned}$$
  are satisfied.

We write [semiregular]=R-semiregular,*B*-semiregular. If $\mathfrak {M}$ is [semiregular] then $\mathcal {E}^{[ \mathfrak {M} ]} ( \Omega )$ contains $\mathcal {C}^\omega (\Omega )$ by (3.2) and is closed under derivation. In fact, $\mathcal {E}^{[ \mathfrak {M} ]} ( \Omega )$ is closed under real-analytic mappings, cf. [16].

We now recall the microlocalization of the concept of ultradifferentiability from [16]

### Definition 3.4

Let $\mathfrak {M}$ be a weight matrix and $u\in \mathcal {D}^\prime (\Omega )$.

1. *u* is microlocally ultradifferentiable of class $\{\mathfrak {M}\}$ at $(x_0,\xi _0)\in T^*\Omega \!\setminus \!\{0\}=\Omega \times \mathbb {R}^n\!\setminus \!\{0\}$ if there exists a neighborhood *V* of $x_0$, a conic neigborhood Γ of $\xi _0$ and a bounded sequence $(u_j)_j\subseteq \mathcal {E}^\prime (\Omega )$ with $u_j\vert _V=u\vert _V$ such that for some $\textbf{M}\in \mathfrak {M}$ and some q>0 we have that
  3.5 $$\begin{aligned} \sup _{\begin{array}{c} \xi \in \Gamma \\ j\in \mathbb {Z}_+ \end{array}}\frac{{|\xi |}^j|\hat{u}_j(\xi )|}{q^j M_j}<\infty . \end{aligned}$$
2. *u* is microlocally ultradifferentiable of class $(\mathfrak {M})$ at $(x_0,\xi _0)$ if there are a neighborhood *V* of $x_0$, a conic neighborhood Γ of $\xi _0$ and a bounded sequence $(u_j)_j\subseteq \mathcal {E}^\prime (\Omega )$ with $u_j\vert _V=u\vert _V$ such that for all $\textbf{M}\in \mathfrak {M}$ and all q>0 the estimate (3.5) holds.

We set

The basic properties of ${{\,\textrm{WF}\,}}_{[\mathfrak {M}]}u$ (cf. [16, Proposition 5.4]) are

### Proposition 3.5

Let $\mathfrak {M}$ be a weight matrix and $u\in \mathcal {D}^\prime (\Omega )$.

1. ${{\,\textrm{WF}\,}}_{[\mathfrak {M}]}u$ is a conic and closed subset of .
2. $${{\,\textrm{WF}\,}}_{\{\mathfrak {M}\}}u\subseteq {{\,\textrm{WF}\,}}_{(\mathfrak {M})}u$$.
3. If $\mathfrak {M}$ is [semiregular] then
  $$\begin{aligned} \pi _1\left( {{\,\textrm{WF}\,}}_{[\mathfrak {M}]}u\right) ={{\,\mathrm{sing\, supp}\,}}_{[\mathfrak {M}]}u. \end{aligned}$$
4. If $\mathfrak {M}$ satisfies (3.2) then ${{\,\textrm{WF}\,}}u\subseteq {{\,\textrm{WF}\,}}_{[\mathfrak {M}]}u\subseteq {{\,\textrm{WF}\,}}_a u$.
5. If $\mathfrak {M}$ is [semiregular] and *P* is a linear differential operator with coefficients in $\mathcal {E}^{[ \mathfrak {M} ]} ( \Omega )$ then ${{\,\textrm{WF}\,}}_{[\mathfrak {M}]}Pu\subseteq {{\,\textrm{WF}\,}}_{[\mathfrak {M}]}u$.

Following the arguments in [23, Section 8.6] we can prove the microlocal elliptic regularity theorem in $\mathcal {E}^{[\mathfrak {M}]}$ for analytic differential operators:

### Theorem 3.6

Let *P* a differential operator with analytic coefficients in Ω and $\mathfrak {M}$ be a [semiregular] weight sequence. Then

$$\begin{aligned} {{\,\textrm{WF}\,}}_{[\mathfrak {M}]} u\subseteq {{\,\textrm{WF}\,}}_{[\mathfrak {M}]}Pu\cup {{\,\textrm{Char}\,}}P \end{aligned}$$

for all $$u\in \mathcal {D}^\prime (\Omega )$$.

For a more general version for operators with ultradifferentiable coefficients see [16, Section 7]. As pointed out above we restrict ourselves to analytic differential operators. Nevertheless, for our purposes we still need to extend the microlocal theory presented in [16] by generalizing some statements from [23] which have not been considered in [16].

We recall that the tensor product of two distributions $u\in \mathcal {D}^\prime (U)$ and $v\in \mathcal {D}^\prime (V)$ ($U\subseteq \mathbb {R}^m$, $V\subseteq \mathbb {R}^n$ open), denoted by u⊗v, is defined by

$$\begin{aligned} \langle u\otimes v,\varphi \otimes \psi \rangle =\langle u,\varphi \rangle \cdot \langle v,\psi \rangle \end{aligned}$$

where $\varphi \in \mathcal {C}^\infty _0(U)$ and $\psi \in \mathcal {C}^\infty _0(V)$, cf. [23, Chapter 5].

### Theorem 3.7

Let $U\subseteq \mathbb {R}^m$ and $V\subseteq \mathbb {R}^n$ be open sets and $\mathfrak {M}$ be a [semiregular] weight matrix. Then

$$\begin{aligned} \begin{aligned} {{\,\textrm{WF}\,}}_{[\mathfrak {M}]}(u\otimes v)&\subseteq \left( {{\,\textrm{WF}\,}}_{[\mathfrak {M}]}u\times {{\,\textrm{WF}\,}}_{[\mathfrak {M}]}v\right) \cup \left( \left( {{\,\textrm{supp}\,}}u\times \{0\}\right) \times {{\,\textrm{WF}\,}}_{[\mathfrak {M}]}v\right) \\&\qquad \cup \left( {{\,\textrm{WF}\,}}_{[\mathfrak {M}]}u\times \left( \{0\}\times {{\,\textrm{supp}\,}}v\right) \right) . \end{aligned} \end{aligned}$$

### Proof

Since ${{\,\mathrm{sing\, supp}\,}}_{[\mathfrak {M}]} u\subseteq {{\,\textrm{supp}\,}}u$ it is enough to show that ifthen $(x_0,\xi _0,y_0,\eta _0)\notin {{\,\textrm{WF}\,}}_{[\mathfrak {M}]}(u\otimes v)$.

If $x_0\notin {{\,\textrm{supp}\,}}u$ then there is a neighborhood $U^\prime \subseteq U$ of $x_0$ such that $u\vert _{U^\prime }\equiv 0$. It follows that

$$\begin{aligned} (u\otimes v)\vert _{U^\prime \times V}\equiv 0 \end{aligned}$$

and thus $(x_0,\xi _0,y_0,\eta _0)\notin {{\,\textrm{WF}\,}}_{[\mathfrak {M}]}(u\otimes v)$. Analogously we see that if $y_0\notin {{\,\textrm{supp}\,}}v$ then $(x_0,\xi _0,y_0,\eta _0)\notin {{\,\textrm{WF}\,}}_{[\mathfrak {M}]}(u\otimes v)$.

Now, assume that $(x_0,\xi _0)\notin {{\,\textrm{WF}\,}}_{(\mathfrak {M})} u$. Then there are a neighborhood $U_1$ of $x_0$, a conic neighborhood $\Gamma ^\prime$ of $\xi _0$ and a bounded sequence $(u_j)_j\subseteq \mathcal {E}^\prime (U)$ with $u_j\vert _{U_1}=u\vert _{U_1}$ such that for all $\textbf{M}\in \mathfrak {M}$ and every h>0 there is a constant C>0 such that

$$\begin{aligned} {|\xi |}^j|\hat{u}_j(\xi )|\le Ch^jM_j \end{aligned}$$

for all $\xi \in \Gamma ^\prime$ and $j\in \mathbb {Z}_+$.

Now choose a test function $\psi \in \mathcal {C}^\infty _0(V)$ such that $\psi \vert _{V_1}\equiv 1$ for some neighborhood of $y_0$. Then $v_0=\psi v\in \mathcal {E}^\prime (V)$ and $w_j=u_j\otimes v_0\in \mathcal {E}^\prime (U\times V)$ is a bounded sequence with

$$\begin{aligned} w_j\vert _{U_1\times V_1}=(u\otimes v)\vert _{U_1\times V_1}. \end{aligned}$$

Furthermore, we know that there are some $d\in \mathbb {Z}_+$ and $C_0>0$ such that

$$\begin{aligned} |\hat{v}_0(\eta )|\le C_0(1+|\eta |)^d\qquad \;\,\forall \,\eta \in \mathbb {R}^n. \end{aligned}$$

For the tensor product we have that $\hat{w}_j(\xi ,\eta )=\hat{u}_j(\xi )\hat{v}_0(\eta )$. We continue by choosing a conic neighborhood $\Gamma \subseteq \mathbb {R}^{m+n}$ of $(\xi _0,\eta _0)$ such that $\pi _1\Gamma \subseteq \Gamma ^\prime \subseteq \mathbb {R}^m$. Then it follows that

$$\begin{aligned} \begin{aligned} |(\xi ,\eta )|^j |\hat{w}_j(\xi ,\eta )|&=|(\xi ,\eta )|^j |\hat{u}_j(\xi )| |\hat{v}_j(\eta )|\\&\le C_0Ch^{j+d} M_{j+d}\sup _{\begin{array}{c} \xi ^\prime \in \pi _1(\Gamma )\\ \eta ^\prime \in \pi _2(\Gamma ) \end{array}} \frac{|(\xi ^\prime ,\eta ^\prime )|^j(1+|\eta ^\prime |)^d}{|\xi ^\prime |^{j+d}} \end{aligned} \end{aligned}$$

for $(\xi ,\eta )\in \Gamma$. Now we set  and . Then

$$\begin{aligned} \begin{aligned} \sup _{\begin{array}{c} \xi ^\prime \in \pi _1(\Gamma )\\ \eta ^\prime \in \pi _2(\Gamma ) \end{array}} \frac{|(\xi ^\prime ,\eta ^\prime )|^j(1+|\eta ^\prime |)^d}{|\xi ^\prime |^{j+d}}&\le \sup _{\begin{array}{c} \xi ^\prime \in \Gamma _1\\ \eta ^\prime \in \Gamma _2\\ \rho >1 \end{array}} \frac{|(\rho \xi ^\prime ,\rho \eta ^\prime |^j(1+|\rho \eta ^\prime |)^d}{|\rho \xi ^\prime |^{j+d}}\\&\le \rho ^{j+d-j-d} \sup _{\begin{array}{c} \xi ^\prime \in \Gamma _1\\ \eta ^\prime \in \Gamma _2 \end{array}} |(\xi ^\prime ,\eta ^\prime )|^j\le \tau ^j \end{aligned} \end{aligned}$$

for some τ>0. Since *d* only depends on *v* we can apply (3.4) and conclude that for each $\textbf{N}\in \mathfrak {M}$ there are some $\textbf{M}\in \mathfrak {M}$ and a constant γ>0 such that $M_{j+d}\le \gamma ^{j+d+1}N_j$ for all $j\in \mathbb {Z}_+$. Therefore we have shown that there is a conic neighborhood $\Gamma ^\prime$ of $(\xi _0,\eta _0)$ such that for all $\textbf{N}\in \mathfrak {M}$ and all h>0 we have that

$$\begin{aligned} \sup _{\begin{array}{c} (\xi ,\eta )\in \Gamma ^\prime \\ j\in \mathbb {Z}_+ \end{array}} \frac{|(\xi ,\eta )|^j|\hat{w}_j(\xi ,\eta )|}{h^jN_j}<\infty . \end{aligned}$$

Hence $(x_0,\eta _0,y_0,\eta _0)\notin {{\,\textrm{WF}\,}}_{(\mathfrak {M})} (u\otimes v)$. The Roumieu case follows similarly.

If $(y_0,\eta _0)\notin {{\,\textrm{WF}\,}}_{[\mathfrak {M}]}u$ and $\xi _0\ne 0$ we can argue analogously. □

For later reference we recall from [16] some facts. To begin with let

$$\begin{aligned} I(\xi )&=\int _{|\omega |=1}\! e^{\omega \xi }\,d\omega ,\\ \tilde{K}(z)&=(2\pi )^{-n}\int \! e^{iz\xi }/I(\xi )\,d\xi . \end{aligned}$$

### Theorem 3.8

([16, Theorem 5.7]) If $u\in \mathcal {S}^\prime (\mathbb {R}^n)$ and $U=\tilde{K}*u$, then *U* is analytic in  and there exist *C*, *a*, *b* such that

3.6 $$\begin{aligned} | U(z)|\le C\bigl (1+| z|\bigr )^{a}\bigl (1-|{{\,\textrm{Im}\,}}z|)^{-b},\quad z\in X. \end{aligned}$$

The boundary values $U(\,\cdot \,+i\omega )$ are continuous functions of $\omega \in S^{n-1}$ with values in $\mathcal {S}^\prime (\mathbb {R}^n)$, and

3.7 $$\begin{aligned} \langle u,\varphi \rangle =\int _{S^{n-1}}\!\langle U(\cdot +i\omega ),\varphi \rangle \,d\omega ,\quad \varphi \in \mathcal {S}. \end{aligned}$$

On the other hand, if *U* is given satisfying (3.6), then the formula (3.7) defines a distribution $u\in \mathcal {S}^\prime$ with U=K∗u.

For all [semiregular] weight matrices $\mathfrak {M}$ we have

$$\begin{aligned} \bigl (\mathbb {R}^n\times S^{n-1}\bigr )\cap {{\,\textrm{WF}\,}}_{[\mathfrak {M}]} u= \bigl \{(x,\omega ) : |\omega |=1,\, U\text { is not in }\mathcal {E}^{[\mathfrak {M}]} \text { at } x-i\omega \bigr \}. \end{aligned}$$

Here *U* being in $\mathcal {E}^{[\mathfrak {M}]}$ at x-iω means that there is a neighborhood *V* of x-iω in $\mathbb {C}^n$ such that the derivatives $\partial _z^\alpha U$ satisfy the defining estimates of $\mathcal {E}^{[\mathfrak {M}]}$ in *V*. Theorem 3.8 follows from a straightforward modification of the proof of [23, Theorem 8.4.11] using the [semiregularity] of $\mathfrak {M}$. The same applies to the following corollary (cf. [23, Corollary 8.4.13]).

### Corollary 3.9

([16, Corollary 5.8]) Let $\Gamma ^1,\dotsc \Gamma ^N\subseteq \mathbb {R}^n\!\setminus \!\{0\}$ be closed cones such that $\bigcup _j\Gamma ^j=\mathbb {R}^n\!\setminus \!\{0\}$. Any $u\in \mathcal {S}^\prime (\mathbb {R}^n)$ can be written $u=\sum u_j$, where $u_j\in \mathcal {S}^\prime$ and

3.8 $$\begin{aligned} {{\,\textrm{WF}\,}}_{[\mathfrak {M}]}u_j\subseteq {{\,\textrm{WF}\,}}_{[\mathfrak {M}]}u\cap \bigl (\mathbb {R}^n\times \Gamma ^j\bigr ). \end{aligned}$$

If $u=\sum u^\prime _j$ is another such decomposition, then $u^\prime _j=u_j+\sum _k u_{jk}$, where $u_{jk}\in \mathcal {S}^\prime$, $u_{jk}=-u_{kj}$ and

$$\begin{aligned} {{\,\textrm{WF}\,}}_{[\mathfrak {M}]}u_{jk}\subseteq {{\,\textrm{WF}\,}}_{[\mathfrak {M}]}u\cap \bigl (\mathbb {R}^n\times \bigl (\Gamma ^j\cap \Gamma ^k\bigr )\bigr ). \end{aligned}$$

The next theorem generalizes [23, Theorem 8.4.15]; it suffices to follow the arguments in [23] while taking the above statements into account; recall that  denotes the dual cone of Γ. Moreover a holomorphic function $F\in \mathcal {O}(V+i\Gamma _r)$ is said to be of slow growth if

### Corollary 3.10

Let $\mathfrak {M}$ be a [semiregular] weight matrix. Let $u\in \mathcal {D}^\prime (\Omega )$ and $(x_0,\xi _0)\in T^*\Omega \setminus \{0\}$. Then $(x_0,\xi _0)\notin {{\,\textrm{WF}\,}}_{[\mathfrak {M}]} u$ if and only if there exists a neighborhood *V* of $x_0$, $f\in \mathcal {E}^{[\mathfrak {M}]}(V)$, open cones $\Gamma ^1,\dotsc ,\Gamma ^q$ with the property $\xi _0\Gamma ^j<0$ for all *j*, and holomorphic functions $F_j\in \mathcal {O}(\Omega +i\Gamma ^j_{\delta })$ of slow growth such that

$$\begin{aligned} u\vert _V=f+\sum _{j=1}^qb_{\Gamma ^j}F_j. \end{aligned}$$

In [23] the pullback of distributions with real analytic mappings was defined. Since $\mathcal {E}^{[\mathfrak {M}]}$ is stable by pullback with real analytic mappings, see [16, Theorem 2.9], we can follow the proof of [23, Theorem 8.5.1] to obtain the following statement:

### Theorem 3.11

([16, Theorem 5.11]) Let $\mathfrak {M}$ be a [semiregular] weight matrix. Let $F:\Omega _1\rightarrow \Omega _2$ be a real analytic mapping, where $\Omega _i \in \mathbb {R}^{n_i}$ are open. If $u\in \mathcal {D}^\prime (\Omega _2)$ and $N_F\cap {{\,\textrm{WF}\,}}_{[\mathfrak {M}]}u=\emptyset$, then

$$\begin{aligned} {{\,\textrm{WF}\,}}_{[\mathfrak {M}]}\left( F^*u\right) \subseteq F^*\left( {{\,\textrm{WF}\,}}_{[\mathfrak {M}]}u\right) . \end{aligned}$$

Here  is the set of normals of *F*.

### Theorem 3.12

Let $\mathfrak {M}$ be a [semiregular] weight matrix. If $u,v\in \mathcal {D}^\prime (\Omega )$ are such that $(x,\xi )\in {{\,\textrm{WF}\,}}_{[\mathfrak {M}]}u$ implies that $(x,-\xi )\notin {{\,\textrm{WF}\,}}_{[\mathfrak {M}]}v$ then the product *uv* is well defined as a distribution in $D^\prime (\Omega )$ and

### Proof

That *uv* is well-defined follows from [23, Theorem 8.2.10]. There the product *uv* is defined as

$$\begin{aligned} \delta ^*(u\otimes v) \end{aligned}$$

where δ(x)=(x,x) is the diagonal map from Ω to $\Omega \times \Omega$. The statement follows from the proof of [23, Theorem 8.2.10] when we apply Theorem 3.7 and Theorem 3.11. □

### Proposition 3.13

Let $u\in \mathcal {E}^\prime (\mathbb {R}^{n+m})$ and denote the variables of $\mathbb {R}^{n+m}$ by (*x*, *y*). We define

$$\begin{aligned} u_1=\int _{\mathbb {R}^m}\!u(\,.\,,y)\,dy\in \mathcal {E}^\prime (\mathbb {R}^n). \end{aligned}$$

(More precisely, $\langle u_1,\phi \rangle =\langle u,\phi \otimes 1\rangle$). Thenfor all [semiregular] weight matrices $\mathfrak {M}$.

### Proof

Using the notation of [16, Theorem 5.7] we have for $\phi \in \mathcal {C}^\infty _0(\mathbb {R}^n)$ and $\psi \in \mathcal {C}^\infty _0(\mathbb {R}^m)$ that

$$\begin{aligned} \langle u,\phi \otimes \psi \rangle =\int _{|\omega |=1}\!\langle U(\,.\,+i\omega ),\phi \otimes \psi \rangle \,d\omega . \end{aligned}$$

Take $\psi (y)=\chi (\delta y)$ where χ is a test function that equals 1 on the unit ball, and let $\delta \rightarrow 1$. Since *U* is exponentially decreasing at infinity it follows that

$$\begin{aligned} \langle u_1,\phi \rangle =\int _{|\omega |=1}\!\langle U(\,.\,+i\omega ),\phi \otimes 1\rangle \,d\omega =\int _{|\tau |=1}\! U_1(\, .\,+i\sigma ),\phi \rangle \,d\sigma \end{aligned}$$

where

$$\begin{aligned} U_1(\zeta )=\int \! U(\zeta ,y)\,dy=\int \! U(\zeta ,y+\tau )\,dy,\qquad \quad |{{\,\textrm{Im}\,}}\zeta |^2+|\tau |^2<1, \end{aligned}$$

is an analytic function when $|{{\,\textrm{Im}\,}}\zeta |<1$ which is bounded by $C(1-|{{\,\textrm{Im}\,}}\zeta |)^{-N}$. If |σ|=1 and $(x,y,\sigma ,0)\notin {{\,\textrm{WF}\,}}_{[\mathfrak {M}]} u$ for all $y\in \mathbb {R}^m$ then $U_1\in \mathcal {E}_{[\mathfrak {M}]}$ at x-iσ by Theorem 3.8. Hence the straightforward generalization of [23, Lemma 8.4.12] implies $(x,\sigma )\notin {{\,\textrm{WF}\,}}_{[\mathfrak {M}]}u_1$. □

For the next statement we need more definitions: Let $U\subseteq \mathbb {R}^m$ and $V\in \mathbb {R}^n$ be open sets and $K\in \mathcal {D}^\prime (U\times V)$. We setIt is clear that *K* defines a mapping $\mathcal {K}:\mathcal {C}^\infty _0(V)\rightarrow \mathcal {D}^\prime (U)$. Unter certain assumptions on the wavefront sets of *K* and $u\in \mathcal {D}^\prime (V)$ it is possible to extend $\mathcal {K}$ to make sense of $\mathcal {K}u$, cf. [23, Theorem 8.2.13 & Theorem 8.5.5]. The following Theorem is the main statement of this section and is a direct generalization of [23, Theorem 8.5.5].

### Theorem 3.14

If $u\in \mathcal {E}^\prime (V)$ and ${{\,\textrm{WF}\,}}_{[\mathfrak {M}]}u\cap {{\,\textrm{WF}\,}}_{[\mathfrak {M}]}^\prime (K)_V=\emptyset$ then $\mathcal {K}u\in \mathcal {D}^\prime (U)$ is well-defined

$$\begin{aligned} \begin{aligned} {{\,\textrm{WF}\,}}_{[\mathfrak {M}]}(\mathcal {K}u)&\subseteq {{\,\textrm{WF}\,}}_{[\mathfrak {M}]}(K)_U\cup \left( {{\,\textrm{WF}\,}}_{[\mathfrak {M}]}^\prime (K)\circ {{\,\textrm{WF}\,}}_{[\mathfrak {M}]}u\right) \\&={{\,\textrm{WF}\,}}_{[\mathfrak {M}]}(K)_U\cup \Bigl \{(x,\xi )\in U\times \mathbb {R}^n\setminus \{0\}\;:\\&\qquad \qquad \qquad (x,y,\xi ,-\eta )\in {{\,\textrm{WF}\,}}_{[\mathfrak {M}]}(K)\text { for some }(x,\eta )\in {{\,\textrm{WF}\,}}_{[\mathfrak {M}]}u\Bigr \}. \end{aligned} \end{aligned}$$

### Proof

By Theorem 3.7 we have thatThence $K_u=K(1\otimes u)$ is well-defined and Theorem 3.12 givesNow $\langle \mathcal {K}u,\psi \rangle =\langle K_u,\psi \otimes 1\rangle$ by the definition of $\mathcal {K}$ in [23, Theorem 8.2.13]. Therefore the assertion follows from Proposition 3.13. □

### Remark 3.15

If $\mathfrak {M}=\{\textbf{M}\}$ consists of a single weight sequence then in the Roumieu case we just recover the results of [23, Section 8.5]. In the case of weight functions we need a little more work, cf. [34]:

If ω is a weight function then we associate to ω a weight matrix  by setting

$$\begin{aligned} W_j^\lambda =\exp \left( \frac{1}{\lambda }\varphi _\omega ^*(\lambda j)\right) . \end{aligned}$$

Then $\mathfrak {W}$ satisfies the following properties

$$\begin{aligned} \;\,\forall \,\lambda>0\,\;\,\forall \,j_1,j_2\in \mathbb {Z}_+\quad W^{\lambda }_{j_1+j_2}\le W_{j_1}^{2\lambda } W_{j_2}^{2\lambda }\\ \;\,\forall \,h\ge 1 \,\;\,\exists \,A\ge 1\, \;\,\forall \,\lambda >0\, \;\,\exists \,D\ge 1 \,\;\,\forall \,j\in \mathbb {Z}_+:\quad h^jW_j^\lambda \le DW_j^{A\lambda }. \end{aligned}$$

Thus $\mathcal {E}^{[ \omega ]} ( \Omega )$ and $\mathcal {E}^{[ \mathfrak {W} ]} ( \Omega )$ are isomorphic as topological vector spaces. Furthermore $\mathfrak {W}$ is *R*- and *B*-semiregular if ω(t)=o(t), $t\rightarrow \infty$. Finally it is easy to see that ${{\,\textrm{WF}\,}}_{[\mathfrak {W}]}u={{\,\textrm{WF}\,}}_{[\omega ]}u$ where ${{\,\textrm{WF}\,}}_{[\omega ]}u$ is the wavefront set associated to $\mathcal {E}^{[ \omega ]} ( \Omega )$ defined in [1].

We close this section by presenting a few applications of the main Theorem 3.14. First, we observe that, by using Theorem 3.14 and Theorem 3.11, we can easily extend the arguments from [23, p. 270] and obtain the following statement regarding the convolution of distributions.

### Corollary 3.16

Let $k\in \mathcal {D}^\prime (\mathbb {R}^n)$ and $u\in \mathcal {E}^\prime (\mathbb {R}^n)$. Then

We apply Corollary 3.16 to the asymptotically analytic cutoff multipliers from [42, Section 17.3]. More precisely, given two open cones $\Gamma ,\Gamma ^\prime \subseteq \mathbb {R}^n\setminus \{0\}$ with $\Gamma ^\prime \cap S^{n-1}\Subset \Gamma \cap S^{n-1}\ne S^{n-1}$ (We assume here that n≥2) and R>0 there is a smooth function $g_R\in \mathcal {C}^\infty (\mathbb {C}\!\setminus \! i\mathbb {R}^n)$ with the following properties (cf. [42, Lemma 17.3.2]):

- $$\;\,\forall \,\xi \in \mathbb {R}^n\setminus \{0\}:\quad 0\le g_R(\xi )\le 1$$
- $$\;\,\forall \,\xi \in \mathbb {R}^n\setminus \{0\},\;\,\forall \,\alpha \in \mathbb {Z}_+^n,\;{|\xi |}\ge 2R{|\alpha |}:\quad |D^\alpha g_R(\xi )|\le 2(C_1/R)^{{|\alpha |}}$$
- $$\;\,\forall \,\zeta =\xi +i\eta \in \mathbb {C}^n\!\setminus \!i\mathbb {R}^n$$:
  - If $$\xi \in \Gamma ^\prime $$ then $$g_R(\xi )=1$$.
  - If $$\xi \notin \Gamma $$ then $$g_R(\xi )=0$$.
- $$\;\,\forall \,\zeta =\xi +i\eta \in \mathbb {C}^n\!\setminus \!i\mathbb {R}^n$$:
  $$\begin{aligned} |g_R(\zeta )|\le \exp \left( C_1\frac{|\eta |}{R}\right) \\ |\partial _{\zeta }g_R(\zeta )|\le C_2\exp \left( (C_3|\eta |-|\xi |)/R\right) \end{aligned}$$

The constants $C_1$, $C_2$ and $C_3$ do not depend on *R*.

If we denote by $G_R=\mathcal {F}^{-1}(g_R)$ the inverse Fourier transform of $g_R$ then we obtain from [42, Lemma 17.3.6] that ${{\,\textrm{WF}\,}}_a G_R\subseteq \mathbb {R}^n\times \overline{\Gamma }$ since $G_R*\delta =G_R$. Moreover, $G_R$ is analytic in the region |x|>C/R where *C* is a constant independent of *R* cf. [42, Proposition 17.3.3]. We obtain thatfor any [semiregular] weight matrix. Following the presentation in [42, Section 17.3] we write

$$\begin{aligned} g_R(D)u=G_R*u,\qquad u\in \mathcal {E}^\prime (\mathbb {R}^n). \end{aligned}$$

### Proposition 3.17

Let $u\in \mathcal {E}^\prime (\mathbb {R}^n)$, $(x_0,\xi _0)\in \mathbb {R}^n \times \mathbb {R}^n\setminus \{0\}$ and $\mathfrak {M}$ be a [semiregular] weight sequence. Then $(x_0,\xi _0)\notin {{\,\textrm{WF}\,}}_{[\mathfrak {M}]}u$ if and only if there are an open neighborhood of $x_0$, two open cones $\xi _0\in \Gamma ^\prime \Subset \Gamma \subsetneq \mathbb {R}^n\setminus \{0\}$ and some R>0 such that $g_R(D)u\vert _U\in \mathcal {E}^{[ \mathfrak {M} ]} ( U )$.

### Proof

If $(x_0,\xi _0)\notin {{\,\textrm{WF}\,}}_{[\mathfrak {M}]} u$ then there is an open neighborhood *V* of $x_0$ and some open convex cone $\xi _0\in F\subsetneq \mathbb {R}^n\setminus \{0\}$ such that ${{\,\textrm{WF}\,}}_{[\mathfrak {M}]}u\cap V\times F\ne \emptyset$. We choose two open convex cones $\Gamma ,\Gamma ^\prime$ such that $\xi _0 \in \Gamma ^\prime \Subset \Gamma \Subset F$. For *R* to be determined later we take the multiplier $g_R$ from above which is associated to the cones Γ and $\Gamma ^\prime$. We have shown that . If we choose R>0 such that  then it follows that there is a neighborhood *W* of $x_0$ such thatThe other direction can be proven in the same way as the sufficiency part of the proof of [42, Proposition 17.3.7]: If $g_R$ is an multiplier of the above form for the cones $\Gamma ^\prime \Subset \Gamma$, then the same is true for $1-g_R$ with respect to the cones $\emptyset \ne \mathbb {R}^n\setminus \Gamma \Subset \mathbb {R}^n\setminus \Gamma ^\prime \subsetneq \mathbb {R}^n\setminus \{0\}$. Thence

$$\begin{aligned} {{\,\textrm{WF}\,}}_{[\mathfrak {M}]} (1-g_R(D))u\cap \mathbb {R}^n\times \Gamma ^\prime&=\emptyset \end{aligned}$$

and thus

$$\begin{aligned} {{\,\textrm{WF}\,}}_{[\mathfrak {M}]} u\cap \mathbb {R}^n\times \Gamma ^\prime&=\emptyset . \end{aligned}$$

□

Finally we are now able to prove Theorem 2.10 which will follow from the combination of [13, Theorem 5] and the following statement:

### Theorem 3.18

Let k≥2 be an even integer and $\mathfrak {M}$ be a [semiregular] weight matrix. If $p_0=(x_0,0;\xi _0,0)\in T^*\mathbb {R}^2\!\setminus \!\{0\}$ then the following statements are equivalent:

1. $Q_\lambda$ is $[\mathfrak {M}]$-hypoelliptic at $p_0$, i.e. for any distribution *u* defined near $(x_0,0)$ we have that $p\notin {{\,\textrm{WF}\,}}_{[\mathfrak {M}]} Q_\lambda u$ implies $p\notin {{\,\textrm{WF}\,}}_{[\mathfrak {M}]} u$
2. $$\lambda \notin \Lambda _k$$

### Proof

Given $\lambda \in \Lambda _k$ and $p_0=(x_0,0;\xi _0,0)$ then according to [13, Lemma 1] there exists a distribution $u\in \mathcal {D}^\prime (\mathbb {R}^n)$ such that $Q_\lambda u=0$ andThus Proposition 3.5(4) gives thatfor any [semiregular] weight matrix $\mathfrak {M}$. Therefore the implication (1)⇒(2) is proven. If (2) holds then in [13, Section 5] a left parametrix for $Q_\lambda$ is constructed:

$$\begin{aligned} \mathcal {K}Q_\lambda ={{\,\textrm{Id}\,}}+\mathcal {R}\end{aligned}$$

in $\mathcal {S}(\mathbb {R}^2)$ and the kernels *K* and *R* of $\mathcal {K}$ and $\mathcal {R}$, respectively, satisfysee [13, equations (33) & (35)]. Now (1) follows from Theorem 3.5(3) and Theorem 3.14. □

## Analytic pseudodifferential operators and ultradifferentiable microdistributions

An obvious application of Theorem 3.14 would be, for example, that *s*-Gevrey pseudodifferential operators (for a definition and details see e.g. [35]) is $[\mathfrak {M}]$-pseudolocal with respect to any ultradifferentiable class $\mathcal {E}^{[\mathfrak {M}]}\supseteq \mathcal {G}^s$, which is closed under differentiation. The next step would be to consider ultradifferentiable pseudodifferential operators but this would involve more conditions on the data of the ultradifferentiable classes than we considered in the section before. Thus we relegate this topic to a forthcoming paper and focus in this paper, as noted in the introduction, on analytic operators, i.e. generally operators with kernels which analytic wavefront sets are “well-behaved”. Such operators include in particular analytic differential operators. For completeness, we have included the following theorem which gives a very large family of operators *A* such that ${{\,\textrm{WF}\,}}_{[\mathfrak {M}]}Au\subseteq {{\,\textrm{WF}\,}}_{[\mathfrak {M}]}u$ for every [semiregular] weight matrix $\mathfrak {M}$ by Theorem 3.14 and Proposition 3.5(4).

### Theorem 4.1

Let $\Omega \subseteq \mathbb {R}^n$ be an open set and $\Phi \in \mathcal {C}^\infty (\Omega \times \mathbb {R}^N\!\setminus \!\{0\})$ with ${{\,\textrm{Im}\,}}\Phi \ge 0$. Suppose that Φ depends holomorphically on the first variable, is homogeneous of degree 1 in the second variable and $\nabla _x\Phi (x,\theta )\ne 0$ on . Furthermore let $a\in \mathcal {C}^\infty (\Omega \times \mathbb {R}^N)$ be such that *a* extends to a holomorphic function in the first variable on some complex neighborhood $\widetilde{\Omega }$ of Ω and *a* satisfies the following estimates: There is some $d\in \mathbb {R}$ such that for each compact set $K\subseteq \widetilde{\Omega }$, and all $\gamma \in \mathbb {Z}_+^N$ there is a constant $C_\gamma >0$ so that

4.1 $$\begin{aligned} |\partial _\theta ^\gamma a(z,\theta )|\le C_\gamma \left( 1+|\theta |\right) ^{d-|\gamma |} \qquad \;\,\forall \,z\in K,\; \;\,\forall \,\theta \in \mathbb {R}^N. \end{aligned}$$

Then the oscillatory integral

$$\begin{aligned}\begin{gathered} A(x)=\int e^{i\Phi (x,\theta )}a(x,\theta )\,d\theta \in \mathcal {D}^\prime (\Omega ) \end{gathered}\end{aligned}$$

exists and

### Proof

First note that if $\psi \in \mathcal {C}^\infty _0(\mathbb {R}^N)$ with $\psi (\theta )=1$ for |θ|<1 then

$$\begin{aligned} h_0(x)= \int \psi (\theta )e^{i\Phi (x,\theta )}a(x,\theta )\,d\theta \end{aligned}$$

extends to a holomorphic function in a neighborhood of Ω. Hence we can w.l.o.g. assumme that a(x,θ) vanishes for |θ|<1.

Now assume that $x_0\in \Omega$ such that $d_\theta \Phi (x_0,\theta )\ne 0$ for all $\theta \in \mathbb {R}^N\!\setminus \!\{0\}$. That means that either $d_\theta {{\,\textrm{Re}\,}}\Phi (x_0,\theta )\ne 0$ or $d_\theta {{\,\textrm{Im}\,}}\Phi (x_0,\theta )\ne 0$ for all θ. In the latter case we have necessarily that ${{\,\textrm{Im}\,}}\Phi (x_0,\theta )>0$ for all $\theta \in \mathbb {R}^N\!\setminus \!\{0\}$. Let $V\subseteq \widetilde{\Omega }$ be a complex neighborhood of $x_0$ such that ${{\,\textrm{Im}\,}}\Phi (z,\vartheta )>0$ for all $z\in \overline{V}$ and $\vartheta \in S^{N-1}$. Then there exists some c>0 such that

$$\begin{aligned} |{{\,\textrm{Im}\,}}\Phi (z,\vartheta )|&>c&\;\,\forall \,\vartheta&\in S^{N-1},\;\;\,\forall \,z\in \overline{V}\\ \end{aligned}$$

and therefore

$$\begin{aligned} |{{\,\textrm{Im}\,}}\Phi (z,\theta )|&>c|\theta |&\;\,\forall \,\theta&\in \mathbb {R}^N\!\setminus \!\{0\},\;\;\,\forall \,z\in \overline{V} \end{aligned}$$

by homogeneity. Thence

$$\begin{aligned} A(z)=\int e^{i\Phi (z,\theta )}a(z,\theta )\,d\theta \end{aligned}$$

is a holomorphic function in *V*.

In the first case we choose again a complex neighborhood $V\subseteq \widetilde{\Omega }$ of $x_0$ such that

4.2 $$\begin{aligned} |\partial _{\theta }\Phi (z,\theta )+2i\varepsilon \theta |> c \qquad \;\,\forall \,z\in \overline{V},\;\;\,\forall \,\theta \in S^{N-1},\;\;\,\forall \,\varepsilon \in \mathbb {R}, \end{aligned}$$

for some constant c>0. For ε>0 we set

$$\begin{aligned} A_\varepsilon (x)=\int \!e^{i\Phi (x,\theta )-\varepsilon |\theta |^2} a(x,\theta )\,d\theta . \end{aligned}$$

By [42, Theorem 8.1.13] $A_\varepsilon \rightarrow A$ in $\mathcal {D}^\prime (V)$. Obviously $A_\varepsilon$ extends to a holomorphic function in $\widetilde{\Omega }$. We follow primarily the arguments presented in [42, Section 18.1]: By integration by parts we have that

$$\begin{aligned} \begin{aligned} A_\varepsilon (z)&= \sum _{j=1}^N\int D_{\theta _j}\left( e^{i\Phi (z,\theta )-\varepsilon |\theta |^2}\right) \frac{a(z,\theta )}{\sum _{k=1}^N\partial _{\theta _k}\Phi (z,\theta )+2i\varepsilon \theta _k}\,d\theta \\&=-\sum _{j=1}\int e^{i\Phi (z,\theta )-\varepsilon |\theta |^2} D_{\theta _j}\left( \frac{a(z,\theta )}{ \sum _{k=1}^N\partial _{\theta _k} \Phi (z,\theta )+2i\varepsilon \theta _k}\right) \,d\theta . \end{aligned} \end{aligned}$$

Set $\Psi (z,\theta )=(\sum _{k=1}^N\partial _{\theta _k} \Phi (z,\theta )+2i\theta _k)^{-1}$. Applying (4.2) we conclude that

$$\begin{aligned} D^\alpha \Psi (z,\theta )=O(|\theta |^{-|\alpha |}),\qquad |\theta |\longrightarrow \infty \end{aligned}$$

Therefore, using also (4.1), we obtain that

$$\begin{aligned} D_{\theta _k}\left( \frac{a(z,\theta )}{\partial _{\theta _k} \Phi (z,\theta )+2i\varepsilon \theta _k}\right) \end{aligned}$$

satisfies also estimates of the form (4.1) with *d* replaced by d-1 for all $z\in \overline{V}$ and every $\varepsilon \in \mathbb {R}$. Iterating this procedure we obtain that

$$\begin{aligned} A_\varepsilon (z)=\int \!e^{i\Phi (z,\theta )-\varepsilon |\theta |^2} a_\varepsilon (z,\theta )\,d\varepsilon ,\qquad \varepsilon >0, \end{aligned}$$

where $a_\varepsilon$, $\varepsilon \in \mathbb {R}$, are functions satisfying estimates of the form (4.1) with *d* replaced by -N-1 and

$$\begin{aligned} \sup _{z\in V}|g_\varepsilon (z,\theta )-g_0(z,\theta )|\le C_\varepsilon (1+|\theta |)^{-N-1},\qquad \theta \in \mathbb {R}^N\!\setminus \!\{0\} \end{aligned}$$

where $C_\varepsilon \rightarrow 0$ for $\varepsilon \rightarrow 0$. Thus

$$\begin{aligned} A_\varepsilon (z)\longrightarrow \int \! e^{i\Phi (z,\theta )}a_0(z,\theta )\,d\theta ,\qquad \varepsilon \rightarrow 0, \end{aligned}$$

uniformly on *K*. Thence ${{\,\mathrm{sing\, supp}\,}}_a A\subseteq \pi _x(\Delta _\Phi )$.

It remains to analyze *A* near a point $x_0\in \Omega$ for which there is some $\theta \in \mathbb {R}^N\!\setminus \!\{0\}$ with $d_\theta \Phi (x,\theta )=0$. We choose a small neighborhood *V* of $x_0$ and definewhich is obviously a closed cone in $\mathbb {R}^N\!\setminus \!\{0\}$.

We consider a covering of $\Delta _V$ by convex, open cones: Let $F_1,\dotsc ,F_\nu$ be open convex cones such that

$$\begin{aligned}\begin{gathered} \Delta _V\subseteq \bigcup _{j=1}^\nu F_j \end{gathered}\end{aligned}$$

and

$$\begin{aligned}\begin{gathered} d_x\Phi (x,\theta )\ne 0,\qquad \;\,\forall \,(x,\theta )\in V\times \bigcup _{j=1}^\nu F_j. \end{gathered}\end{aligned}$$

We choose smooth functions $\chi _j\in \mathcal {C}^\infty (\mathbb {R}^N)$ such that $0\le \chi _j\le 1$, $\chi _j(\theta )=0$ if |θ|<1/2 or if $\theta \notin \ \mathbb {R}^N\setminus F_j$ for all $1\le j\le \nu$ and

$$\begin{aligned} \sum _{j=1}^\nu \chi _j(\theta )=1,\qquad \;\,\forall \,\theta \in \Delta _V,\;|\theta |\ge 1. \end{aligned}$$

We set $\gamma =1-\sum \chi _j$. Thus formally

$$\begin{aligned} \int \!e^{i\Phi (x,\theta )}a(x,\theta )\,d\theta = \int \!e^{i\Phi (x,\theta )}\gamma (\theta )a(x,\theta )\,d\theta +\sum _{j=1}^\nu \int \!e^{i\Phi (x,\delta )}\chi _j(\theta ) a(x,\theta )\,d\theta . \end{aligned}$$

Following the arguments in the first part of the proof we can show that the first integral on the right-hand side defines a holomorphic function in a complex neighborhood of $x_0$.

It remains to look at the oscillatory integral $B_j=\int e^{i\Phi }b_j(x,\theta )\,d\theta$ where we have set $b_j(x,\theta )=\chi _j(\theta )a(x,\theta )$ for $j=1,\dotsc ,\nu$. We begin by considering the Taylor expansion of $\Phi (x+iy,\theta )$ with respect to *y*:

$$\begin{aligned} \Phi (x+iy,\theta )=\Phi (x,\theta )+\nabla _y\Phi (x,\theta )y+ O(|y|^2),\qquad x+iy\in \widetilde{\Omega },\theta \in \mathbb {R}^N\setminus \{0\}. \end{aligned}$$

Since $\Phi (x+iy,\theta )$ is holomorphic in the first variable we have that $\nabla _y\Phi =i\nabla _x\Phi$. Thence

$$\begin{aligned} {{\,\textrm{Im}\,}}\Phi (x+iy,\theta )={{\,\textrm{Im}\,}}\Phi (x,\theta )+\nabla _x{{\,\textrm{Re}\,}}\Phi (x,\theta )y+O(|y|^2) \end{aligned}$$

It follows that

$$\begin{aligned} G_j(x+iy)=\int e^{i\Phi (x+iy,\theta )}b_j(x+iy,\theta )\,d\theta \end{aligned}$$

is holomorphic in the set $\Omega + i\Gamma ^j_\delta$ for δ>0 small enough. Herewith . Obviously $V_j$ and $\Gamma ^j$ are open cones in $\mathbb {R}^n\!\setminus \!\{0\}$ and we write also .

We denote the boundary value (as a hyperfunction in *V*) of $G_j$ by ${{\,\textrm{bv}\,}}G_j$. Recall from [42, Chapter 17] that a representative μ of the restriction ${{\,\textrm{bv}\,}}G_j$ to any open subset $V^\prime \subseteq V$ is given by the limit in $\mathcal {O}^\prime (\mathbb {C}^n)$ of the analytic functionals

$$\begin{aligned} \mathcal {O}(\mathbb {C}^n)\ni \varphi \xrightarrow {\quad \mu _t\quad }\int _{V^\prime +itv}G_j(w)\varphi (w)\,dw,\qquad (1>t\rightarrow +0). \end{aligned}$$

Now observe that, if we denote

$$\begin{aligned} G_j^\varepsilon (z)=\int \!e^{i\Phi (z,\theta )-\varepsilon |\theta |^2} b_j(z,\theta )\,d\theta , \end{aligned}$$

then $G^\varepsilon _j$ converges to $G_j$ in $\mathcal {O}(V+i\Gamma ^j_\delta )$. Hence the analytic functionals

$$\begin{aligned} \mu _t^\varepsilon (\varphi )=\int _{V^\prime +itv}\! G_j(w)\varphi (w)\,dw,\qquad \varphi \in \mathcal {O}(\mathbb {C}^n), \end{aligned}$$

converge to μ in $\mathcal {O}(\mathbb {C}^n)$ for $t,\varepsilon \rightarrow \infty$. On the other hand, the holomorphic functions $G_j^\varepsilon (x+iy)$ converge for y→0 to the smooth function

$$\begin{aligned} B_j^\varepsilon (x)=\int \!e^{i\Phi (x,\theta )-\varepsilon |\theta |^2}b_j(x,\theta )\,d\theta \end{aligned}$$

in $\mathcal {C}(V)$ and $B_j^\varepsilon$ converge to $B_j$ in $\mathcal {D}^\prime (V)$. Now, choose for any $V^\prime \Subset V$ a function $\psi \in \mathcal {C}^\infty _0(V)$ with $\psi \vert _{V^\prime }=1$. It follows that $\psi B_j^\varepsilon (.\,+iy)\rightarrow \psi B$ in $\mathcal {E}^\prime (\mathbb {R}^n)$ for $y,\varepsilon \rightarrow 0$. By the canonical embedding of $\mathcal {E}^\prime (\mathbb {R}^n)$ into $\mathcal {O}^\prime (\mathbb {C}^n)$ we have that $\psi B_j^\varepsilon (.\,+iy)$ converges to ψB in $\mathcal {O}^\prime (\mathbb {C}^n)$. Therefore ${{\,\textrm{bv}\,}}G_j$ coincide with $B_j$ as hyperfunctions in some neighborhood of $x_0$. When we may shrink *V* if necessary, we conclude that $B_j\vert _V$ is the distributional boundary value of $G_j$ (cf. [42, Theorem 7.4.15]) and by [42, Theorem 3.5.5] we have that

$$\begin{aligned} {{\,\textrm{WF}\,}}_a B_j\subseteq V\times (\Gamma ^j)^\circ \end{aligned}$$

where $(\Gamma ^j)^\circ$ is the polar cone of $\Gamma ^j$, i.e.In order to finish the proof note that, if $\xi _0\in \mathbb {R}^n\!\setminus \{0\}$ is such that $(x_0,\xi _0)\notin \Delta _\Phi$ then we can choose the cones $F_j$, $j=1,\dotsc ,\nu$, in such a way that $\xi _0\notin \overline{V_j}$ for all $j=1,\dotsc ,\nu$. Thence $(x_0,\xi _0)\notin {{\,\textrm{WF}\,}}_a A$. □

The class of distributions appearing in Theorem 4.1 includes the distributional kernels of analytic Fourier integral operators, cf. [42]. In fact, it is easy to see that we can follow [42, Section 18.7] in order to microlocalize the distributional kernels, i.e. $\mathbb {R}^N$ is replaced by an open cone in Theorem 4.1 with the obvious modification of $\Delta _\Phi$ etc. Then Theorem 4.1 combined with Theorem 3.14 gives the following statement.

### Theorem 4.2

Let $\Omega _1\subseteq \mathbb {R}^{n_1},\Omega _2\subseteq \mathbb {R}^{n_2}$ be two open sets and $\Gamma \subseteq \mathbb {R}^N\!\setminus \!\{0\}$ an open cone. If Φ is a real-valued real-analytic phase function on $\Omega _1\times \Omega _2\times \Gamma$ then any analytic Fourier integral operator *A* with phase Φ satisfies

4.3 $$\begin{aligned} {{\,\textrm{WF}\,}}_{[\mathfrak {M}]}Au\subseteq \mathfrak {R}_\Phi ({{\,\textrm{WF}\,}}_{[\mathfrak {M}]} u), \qquad \;\,\forall \,u\in \mathcal {E}^\prime (\Omega _2) \end{aligned}$$

for any [semiregular] weight matrix $\mathfrak {M}$, wherefor any subset $E\subseteq T^*\Omega _2\setminus \{0\}$ and $V_{\Phi }:\,\Delta _{\Phi }\rightarrow T^{*}\Omega _1\setminus \{0\}\times T^{*}\Omega _2\setminus \{0\}$ is the map given by $V_{\Phi }(x,y,\theta )=((x,y,d_x \Phi (x,y\theta )),(x,y,-d_y\Phi (x,y,\theta )))$.

In the case of analytic pseudodifferential operators we give some more details following mainly the presentation of [42]: Let $\Omega \subseteq \mathbb {R}^n$ be an open set. Recall that a pseudoanalytic amplitude *a* of order $d\in \mathbb {R}$ on Ω is a smooth function $a\in \mathcal {C}^\infty (\Omega \times \Omega \times \mathbb {R}^n)$ which can be extended to a smooth function on $\Omega _{\mathbb {C}}\times \Omega _{\mathbb {C}}\times \mathbb {R}^n$, where $\Omega _{\mathbb {C}}$ is a neighborhood of Ω in $\mathbb {C}^n$, and *a* is holomorphic with respect to $(z,w)\in \Omega _{\mathbb {C}}\times \Omega _{\mathbb {C}}$. Moreover, for each compact subset $K\subseteq \Omega _{\mathbb {C}}\times \Omega _{\mathbb {C}}$ there are constants C,ρ>0 such that for all $\gamma \in \mathbb {Z}_+^n$, all (z,w)∈K and every $\xi \in \mathbb {R}^n$ with ${|\xi |}\ge \rho \max \{1,|\gamma |\}$ we have that

4.4 $$\begin{aligned} |\partial ^\gamma _{\xi }a(z,w,\xi )|\le C^{|\gamma |+1}|\gamma |!{|\xi |}^{d-|\gamma |}. \end{aligned}$$

We denote the space of pseudoanalytic amplitudes by $S_a(\Omega \times \Omega )$. The analytic pseudodifferential operator $A:\mathcal {E}^\prime (\Omega )\rightarrow \mathcal {D}^\prime (\Omega )$ associated to the amplitude $a\in S_a(\Omega \times \Omega )$ is defined by

$$\begin{aligned} Au(x)=\frac{1}{(2\pi )^n}\int _{\mathbb {R}^n} e^{ix\xi }\left\langle u(y) , a(x,y,\xi )e^{-iy\xi } \right\rangle _y \,d\xi . \end{aligned}$$

The kernel of *A* is the oscillatory integral

4.5 $$\begin{aligned} A(x,y)=\int _{\mathbb {R}^n}\! a(x,y,\xi )e^{i(x-y)\xi }\,d\xi . \end{aligned}$$

We observe that if we consider the phase-function $\Phi (x,y,\theta )=(x-y)\theta$ then we have that $\Delta _{\Phi }=\{(x,y,\theta ):\; x=y\}$ and $d_{(x,y)}\Phi =\theta dx-\theta dy$. Therefore Theorem 3.14 combined with Theorem 4.1 implies that

4.6 $$\begin{aligned} {{\,\textrm{WF}\,}}_{[\mathfrak {M}]}Au\subseteq {{\,\textrm{WF}\,}}_{[\mathfrak {M}]}u,\qquad \;\,\forall \,u\in \mathcal {E}^\prime (\Omega ), \end{aligned}$$

for any analytic pseudodifferential operator *A* and any [semiregular] weight matrix $\mathfrak {M}$.

Following [42, Chapter 17] we can microlocalize the notion of analytic pseudodifferential operators: If $\Omega \subseteq \mathbb {R}^n$ is an open set and $\Gamma \subseteq \mathbb {R}^n\!\setminus \!\{0\}$ is an open cone then a function $a\in \mathcal {C}^\infty (\Omega \times \Omega \times \Gamma )$ is a pseudoanalytic amplitude if the following conditions hold: There is a complex neighborhood $\Omega ^\mathbb {C}$ of Ω such that for $\xi \in \Gamma$ fixed a(x,y,ξ) extends to a holomorphic function on $\Omega ^\mathbb {C}\times \Omega ^\mathbb {C}$. Each derivative $D^\alpha _x D^\beta _y D^\gamma _\xi a$ is bounded near ξ=0 for any $(\alpha ,\beta ,\gamma )\in \mathbb {Z}_+^{3n}$ and, most importantly, *a* satisfies (4.4) for $(x,y,\xi )\in K\times K\times \Gamma$, where *K* is a compact subset of $\Omega ^\mathbb {C}$. We call a continuous operator $A:\mathcal {E}^\prime (\Omega )\rightarrow \mathcal {D}^\prime (\Omega )$ an analytic pseudodifferential operator associated to the open cone $\Gamma \subseteq \mathbb {R}^n \setminus \{0\}$ if there is a pseudoanalytic amplitude on $\Omega \times \Omega \times \Gamma$ such that

$$\begin{aligned} Au(x)=\frac{1}{(2\pi )^n}\int _\Gamma \!e^{ix\xi }\left\langle u(y) , a(x,y,\xi )e^{-iy\xi } \right\rangle _y \,d\xi . \end{aligned}$$

Accordingly the distribution kernel of *A* is given by the oscillatory integral (4.5) with Γ replacing $\mathbb {R}^n$ as the “domain of integration”. It is easily seen that the proof of Theorem 4.1 works also in this case and combined with Theorem 3.14 gives immediately the following statement, where $\Psi ^d_a(\Omega ;\Gamma )$ denotes the space of analytic pseudodifferential operators of order *d* associated to the cone Γ.

### Theorem 4.3

If $$A\in \Psi ^d_a(\Omega ;\Gamma )$$ then

$$\begin{aligned} {{\,\textrm{WF}\,}}_{[\mathfrak {M}]} Au\subseteq {{\,\textrm{WF}\,}}_{[\mathfrak {M}]} u\cap \Omega \times \Gamma \end{aligned}$$

for all $u\in \mathcal {E}^\prime (\Omega )$ and all [semiregular] weight matrices $\mathfrak {M}$.

In particular *A* is $[\mathfrak {M}]$-pseudolocal, i.e. ${{\,\mathrm{sing\, supp}\,}}_{[\mathfrak {M}]} Au\subseteq {{\,\mathrm{sing\, supp}\,}}_{[\mathfrak {M}]} u$ for all $u\in \mathcal {E}^\prime (\Omega )$.

Theorem 4.3 means in particular that, if we transition to sheaves of analytic pseudodifferential operators and analytic microdifferential operators, then the sections of these sheaves behave again well with respect to the ultradifferentiable setting. Before we give more details in this direction we want to give a more precise statement than Theorem 4.3. To this end, we need to introduce the weight function $\omega _\textbf{M}$ associated to a weight sequence $\textbf{M}$, which is given by $\omega _\textbf{M}(0)=0$ and

4.7 $$\begin{aligned} \omega _\textbf{M}(t)=\sup _{k\in \mathbb {Z}_+}\log \frac{t^k}{M_k},\quad t>0. \end{aligned}$$

We notice that $\omega _\textbf{M}$ is a continuous function on [0,∞) such that $\omega _\textbf{M}(t)\rightarrow \infty$ for $t\rightarrow \infty$. In particular, ω(t) grows faster than $\log t^p$ for any integer *p*, cf. [25]. Note also that

4.8 $$\begin{aligned} e^{-\omega _\textbf{M}(t)}=\inf _{k\in \mathbb {Z}_+}\frac{M_k}{t^k},\qquad t>0. \end{aligned}$$

### Definition 4.4

Let $\mathfrak {M}$ be a weight matrix and $a\in S_a(\Omega \times \Omega \times \Gamma )$.

1. The amplitude *a* is $\{\mathfrak {M}\}$-regularizing in some open conic set $V\times W\times \Gamma ^\prime \subseteq \Omega \times \Omega \times \Gamma$ if there are a weight sequence $\textbf{M}\in \mathfrak {M}$ and positive constants *C*, *h* and ρ such that
  4.9 $$\begin{aligned} |D^\alpha _x D^\beta _ya(x,y,\xi )|\le Ch^{|\alpha +\beta |}M_{|\alpha +\beta |}\exp (-\omega _{\textbf{M}}(\rho {|\xi |})),\qquad (x,y,\xi )\in V\times W\times \Gamma . \end{aligned}$$
2. *a* is $(\mathfrak {M})$-regularizing in $V\times W\times \Gamma ^\prime \subseteq \Omega \times \Omega \times \Gamma$ if for every $\textbf{M}\in \mathfrak {M}$ and all h,ρ>0 there is a constant C>0 such that (4.9) holds.

If *a* is the amplitude associated to the pseudodifferential operator *A* then we define the $[\mathfrak {M}]$-essential support ${{\,\textrm{essupp}\,}}_{[\mathfrak {M}]} A$ of *A* in the following way: $(x_0,\xi _0)\notin {{\,\textrm{essupp}\,}}_{[\mathfrak {M}]} A$ if there is a conic neighborhood $V\times W\times \Gamma ^\prime$ of $(x_0,x_0,\xi _0)$ such that *a* is $[\mathfrak {M}]$-regularizing in $V\times W\times \Gamma$.

### Theorem 4.5

Let $A\in \Psi _a(\Omega ;\Gamma )$ and $\mathfrak {M}$ be a [semiregular] weight matrix. Then

$$\begin{aligned} {{\,\textrm{WF}\,}}_{[\mathfrak {M}]} Au\subseteq {{\,\textrm{WF}\,}}_{[\mathfrak {M}]} u\cap {{\,\textrm{essupp}\,}}_{[\mathfrak {M}]} A,\qquad \;\,\forall \,u\in \mathcal {E}^\prime (\Omega ). \end{aligned}$$

### Proof

In view of Theorem 4.3 it suffices to show that $(x_0,\xi _0)\notin {{\,\textrm{essupp}\,}}_{[\mathfrak {M}]} A$ implies $(x_0,x_0,\xi _0,-\xi _0)\notin {{\,\textrm{WF}\,}}_{[\mathfrak {M}]} A$. We return to the proof of Theorem 4.1 with *x* and θ replaced by (*x*, *y*) and ξ, respectively, and set $\Phi (x,y,\xi )=(x-y)\xi$. The proof of Theorem 4.1 gives that given an open neigborhood *U* and a covering $F_j$, $j=1,\dotsc ,\nu$ of open convex cones of Γ we can write

4.10 $$\begin{aligned} A\vert _U= h_0+\sum _{j=1}^\nu {{\,\textrm{bv}\,}}_{F_j} G_j \end{aligned}$$

where $h_0$ is an analytic function in *U* and $G_j$ are holomorphic functions in $U+i\Gamma ^j$ (without loss of generality of slow growth) with . In fact

$$\begin{aligned} G(x+ix^\prime ,y+iy^\prime )=\int e^{i(x+ix^\prime -y-iy^\prime )\xi }\chi (\xi ) a(x+ix^\prime ,y+iy^\prime ,\xi )\,d\xi \end{aligned}$$

where $0\le \chi _j\le 1$ and . If we write $h_j={{\,\textrm{bv}\,}}G_j\in \mathcal {D}^\prime (U)$ then obviously ${{\,\textrm{WF}\,}}_{[\mathfrak {M}]}h_j\subseteq V\times (\Gamma ^j)^\circ$. We observe that (cf. [27, Subsection 7.2])Now let $(x_0,\xi _0)\notin {{\,\textrm{essupp}\,}}_{[\mathfrak {M}]}A$. Then there exist a neighborhood V×W of $(x_0,x_0)$ and a open cone $\Gamma ^\prime$ containing $\xi _0$ such that a(x,y,ξ) is $[\mathfrak {M}]$-regularizing in $V\times W\times \Gamma ^\prime$; w.l.o.g.  $V\times W \subseteq U$. We may also assume that there is some  such that $\xi _0\in F_{j_0}\subseteq \Gamma ^\prime$ and $\xi _0\notin F_j$ for $j\ne j_0$. We have that

4.11 $$\begin{aligned} h_{j_0}(x,y)=\int e^{i(x-y)\xi }\chi (\xi ) a(x,y,\xi )\,d\xi \end{aligned}$$

as an oscillatory integral. However for $(x,y)\in V\times W$ the integral in the right-hand side of (4.11) is absolutely convergent since the integrand can be bounded by an rapidly decreasing function. Because this bound is uniformly for $(x,y)\in V\times W$ we conclude that $h_{j_0}\vert _{V\times W}\in \mathcal {C}^\infty (V\times W)$. We compute the derivatives of $h_{j_0}$:

$$\begin{aligned} \begin{aligned} D^\alpha _x D^\beta _y h_{j_0}(x,y)&=\int \chi (\xi ) D^\alpha _xD^\beta _y \left( e^{i(x-y)\xi }a(x,y,\xi )\right) \,d\xi \\&=\int \chi (\xi )e^{i(x-y)\xi }\sum _{\begin{array}{c} \alpha ^\prime \le \alpha \\ \beta ^\prime \le \beta \end{array}}\left( {\begin{array}{c}\alpha \\ \alpha ^\prime \end{array}}\right) \left( {\begin{array}{c}\beta \\ \beta ^\prime \end{array}}\right) \xi ^{\alpha ^\prime } (-\xi )^{\beta ^\prime } D^{\alpha -\alpha ^\prime }_x D^{\beta -\beta ^\prime }_y a(x,y,\xi )\,d\xi . \end{aligned} \end{aligned}$$

If *A* is $\{\mathfrak {M}\}$-regularizing we can therefore estimate using (4.8) and (4.9) and obtain

$$\begin{aligned} \begin{aligned} |D^\alpha _x D^\beta _y h_{j_0}(x,y)|&\le \int \limits _{|\xi |\ge 1/2} \sum _{\begin{array}{c} \alpha ^\prime \le \alpha \\ \beta ^\prime \le \beta \end{array}}\left( {\begin{array}{c}\alpha \\ \alpha ^\prime \end{array}}\right) \left( {\begin{array}{c}\beta \\ \beta ^\prime \end{array}}\right) |\xi |^{|\alpha ^\prime +\beta ^\prime |} |D^{\alpha -\alpha ^\prime }_x D^{\beta -\beta ^\prime }_y a(x,y,\xi )|\,d\xi \\&\le C\int \limits _{|\xi |\ge 1/2} \sum _{\begin{array}{c} \alpha ^\prime \le \alpha \\ \beta ^\prime \le \beta \end{array}} \left( {\begin{array}{c}\alpha \\ \alpha ^\prime \end{array}}\right) \left( {\begin{array}{c}\beta \\ \beta ^\prime \end{array}}\right) h^{|\alpha +\beta |-|\alpha ^\prime +\beta ^\prime |} M_{|\alpha +\beta |-|\alpha ^\prime +\beta ^\prime |} |\xi |^{|\alpha ^\prime +\beta ^\prime |} e^{(-\omega _\textbf{M}(\rho |\xi |))}\\&\le C\int \limits _{|\xi |\ge 1/2} \sum _{\begin{array}{c} \alpha ^\prime \le \alpha \\ \beta ^\prime \le \beta \end{array}} \left( {\begin{array}{c}\alpha \\ \alpha ^\prime \end{array}}\right) \left( {\begin{array}{c}\beta \\ \beta ^\prime \end{array}}\right) h^{|\alpha +\beta |-|\alpha ^\prime +\beta ^\prime |} M_{|\alpha +\beta |-|\alpha ^\prime +\beta ^\prime |}\times \\&\qquad \qquad \qquad \times M_{|\alpha ^\prime +\beta ^\prime |+n+1} \rho ^{|\alpha ^\prime +\beta ^\prime |+n+1} \int \limits _{|\xi |\ge 1/2}|\xi |^{-n-1}\,d\xi \\&\le C \rho ^{n+1} \left( 2\max \{h,\rho \}\right) ^{|\alpha +\beta |} M_{|\alpha +\beta |+n+1} \int _{|\xi |\ge 1/2}|\xi |\,d\xi . \end{aligned} \end{aligned}$$

Using (3.3) n+1-times we infer that there are a weight sequence $\textbf{M}^\prime \in \mathfrak {M}$, constants $C^\prime ,h^\prime >0$ such that

$$\begin{aligned} \sup _{(x,y)\in V\times W}|D^\alpha _xD^\beta _y h_{j_0}(x,y)|\le C^\prime (h^\prime )^{|\alpha +\beta |}M^\prime _{|\alpha +\beta |}, \qquad \;\,\forall \,(\alpha ,\beta )\in \mathbb {Z}_+^{2n}. \end{aligned}$$

That means in particular that $h_{j_0}\vert _{V\times W}\in \mathcal {E}^{\{ \mathfrak {M} \}} ( V\times W )$ and therefore using (4.10) we conclude that $(x_0,x_0,\xi _0,-\xi _0)\notin {{\,\textrm{WF}\,}}_{\{\mathfrak {M}\}} A$.

In the Beurling case, it is easy to see how to adapt the above proof to show the implication

$$\begin{aligned} (x_0,\xi _0)\notin {{\,\textrm{essupp}\,}}_{(\mathfrak {M})}\Longrightarrow (x_0,x_0,\xi _0,-\xi _0)\notin {{\,\textrm{WF}\,}}_{(\mathfrak {M})}A \end{aligned}$$

if $\mathfrak {M}$ is *B*-semiregular. □

We have seen that the action of analytic differential operators defined on analytic manifolds on semiregular ultradifferentiable classes is well behaved. Theorem 3.14 will show that the same is true for analytic pseudodifferential operators. It will be convenient to first introduce the notion of microdistributions of ultradifferentiable degree. For the definition of microdistributions in the smooth and analytic category we refer to [39] and [42]. The extension to the ultradifferentiable category is straight-forward:

If $\mathfrak {M}$ is a weight matrix and if $\mathcal {U}\subseteq \mathbb {R}^n\times \mathbb {R}^n\!\setminus \!\{0\}$ is a conic open set then we define on $\mathcal {D}^\prime (\pi _1(\mathcal {U}))$ an equivalence relation:

$$\begin{aligned} u\sim _{[\mathfrak {M}]}v\text { in }\mathcal {U}\quad \Longleftrightarrow \quad {{\,\textrm{WF}\,}}_{[\mathfrak {M}]}(u-v)\cap \mathcal {U}=\emptyset . \end{aligned}$$

For open conic sets $\mathcal {V}\subseteq \mathcal {U}\subseteq T^*\mathbb {R}^n\!\setminus \{0\}$ there is a well-defined map

$$\begin{aligned} \rho _{\mathcal {V},\mathcal {U}}:\quad \mathcal {D}^\prime (\pi (\mathcal {U}))/\!\sim _{[\mathfrak {M}]}^{\mathcal {U}}\longrightarrow \mathcal {D}^\prime (\pi (\mathcal {V}))/\!\sim _{[\mathfrak {M}]}^{\mathcal {V}} \end{aligned}$$

induced by the restriction map $\mathcal {D}^\prime (\pi (\mathcal {U}))\rightarrow \mathcal {D}^\prime (\pi (\mathcal {V}))$. Thence $(\mathcal {D}^\prime (\pi (\mathcal {U}))/\!\sim _{[\mathfrak {M}]}^{\mathcal {U}},\rho _{\mathcal {V},\mathcal {U}})$ is a presheaf on $T^*\mathbb {R}^n\!\setminus \!\{0\}$. We denote the resulting sheaf by $\textsf{D}^{micro}_{[\mathfrak {M}]}(\mathbb {R}^n)$ and call it the sheaf of microdistributions of degree $[\mathfrak {M}]$. The stalk of this sheaf at a point $(x,\xi )\in T^*(\mathbb {R}^n)$ is equivalent to $\mathcal {D}^\prime _{x_0}/\sim ^{(x_0,\xi _0)}_{[\mathfrak {M}]}$ where $\mathcal {D}^\prime _{x_0}$ is the set of germs of distributions at $x_0$ and, if $u,v\in \mathcal {D}^\prime _{x_0}$, then

$$\begin{aligned} u \sim _{[\mathfrak {M}]}^{(x_0,\xi _0)} v\quad \Longleftrightarrow \quad (x_0,\xi _0)\notin {{\,\textrm{WF}\,}}_{[\mathfrak {M}]}(u-v). \end{aligned}$$

If we denote the sheaf of (smooth) microdistributions and the sheaf of analytic microdistributions by $\textsf{D}^{micro}(\mathbb {R}^n)$ and $\textsf{D}^{micro}_a(\mathbb {R}^n)$, respectively, then, by Proposition 3.5, there are sheaf morphisms

$$\begin{aligned} \textsf{D}_a^{micro}(\mathbb {R}^n)\longrightarrow \textsf{D}^{micro}_{(\mathfrak {M})}(\mathbb {R}^n)\longrightarrow \textsf{D}^{micro}_{\{\mathfrak {M}\}}(\mathbb {R}^n)\longrightarrow \textsf{D}^{micro}(\mathbb {R}^n) \end{aligned}$$

for any weight matrix $\mathfrak {M}$ satisfying (3.2). Now assume that $\mathfrak {M}$ and $\mathfrak {N}$ are two weight matrices. Then we set

$$\begin{aligned} \mathfrak {N}&\{\preceq \}\mathfrak {M}  &   :\Longleftrightarrow&\;\,\forall \,\textbf{N}\in \mathfrak {N}\;\;\,\exists \,\textbf{M}\in \mathfrak {M}\;\;\,\exists \,h>0\;\;\,\exists \,C>0\quad N_k&\le Ch^kM_k\qquad \;\,\forall \,k\in \mathbb {Z}_+,\\ \mathfrak {N}&(\preceq )\mathfrak {M}  &   :\Longleftrightarrow&\;\,\forall \,\textbf{M}\in \mathfrak {M}\;\;\,\exists \,\textbf{N}\in \mathfrak {N}\; \;\,\exists \,h>0\;\;\,\exists \,C>0\quad N_k&\le Ch^k M_k\qquad \;\,\forall \,k\in \mathbb {Z}_+,\\ \mathfrak {N}&\{\lhd )\mathfrak {M}  &   :\Longleftrightarrow&\;\,\forall \,\textbf{N}\in \mathfrak {N}\;\;\,\forall \,\textbf{M}\in \mathfrak {M}\;\;\,\forall \,h>0\;\;\,\exists \,C>0\quad N_k&\le Ch^kM_k\qquad \;\,\forall \,k\in \mathbb {Z}_+. \end{aligned}$$

If $\mathfrak {N}[\preceq ]\mathfrak {M}$ then ${{\,\textrm{WF}\,}}_{[\mathfrak {M}]}u\subseteq {{\,\textrm{WF}\,}}_{[\mathfrak {N}]}u$ for all $u\in \mathcal {D}^\prime$. Moreover, ${{\,\textrm{WF}\,}}_{(\mathfrak {M})}u\subseteq {{\,\textrm{WF}\,}}_{\{\mathfrak {N}\}}u$ if $\mathfrak {N}\{\lhd )\mathfrak {M}$. Therefore there are sheaf morphisms $\textsf{D}^{micro}_{[\mathfrak {N}]}(\mathbb {R}^n)\rightarrow \textsf{D}^{micro}_{[\mathfrak {M}]}(\mathbb {R}^n)$, when $\mathfrak {N}[\preceq ]\mathfrak {M}$, and $\textsf{D}^{micro}_{\{\mathfrak {N}\}}(\mathbb {R}^n)\rightarrow \textsf{D}^{micro}_{(\mathfrak {M})}(\mathbb {R}^n)$ for $\mathfrak {N}\{\lhd )\mathfrak {M}$.

We denote the space of (continuous) sections of $\textsf{D}_{[\mathfrak {M}]}^{micro}(\mathbb {R}^n)$ over an open conic set $\mathcal {U}\subseteq T^*\mathbb {R}^n$ by $\mathfrak {D}^{micro}_{[\mathfrak {M}]}(\mathcal {U})$. Note that we have an obvious notion of “support” for elements of $\mathfrak {D}^{micro}_{[\mathfrak {M}]}(\mathcal {U})$. Namely, if $(x,\xi )\in \mathcal {U}$ then $(x,\xi )\notin {{\,\textrm{supp}\,}}_{[\mathfrak {M}]} [u]$, $[u]\in \mathfrak {D}^{micro}_{[\mathfrak {M}]}(\mathcal {U})$, if there is a germ *v* of a distribution at $x_0$ such that *v* is a representative of [*u*] at (x,ξ) and $v\sim _{[\mathfrak {M}]} 0$ at (x,ξ). We call an operator $T: \mathfrak {D}^{micro}_{[\mathfrak {N}]}(\mathcal {U})\rightarrow \mathfrak {D}^{micro}_{[\mathfrak {M}]}(\mathcal {U})$ local if

$$\begin{aligned} {{\,\textrm{supp}\,}}_{[\mathfrak {M}]} T[u]\subseteq {{\,\textrm{supp}\,}}_{[\mathfrak {N}]}[u]\qquad \;\,\forall \,[u]\in \mathfrak {D}^{micro}_{[\mathfrak {N}]}(\mathcal {U}). \end{aligned}$$

Obviously we can also introduce similar notions for operators $S:\,\mathfrak {D}^{micro}_{(\mathfrak {N})}\rightarrow \mathfrak {D}^{micro}_{\{\mathfrak {M}\}}$, $R:\,\mathfrak {D}^{micro}_{\{\mathfrak {N}\}}\rightarrow \mathfrak {D}^{micro}_{(\mathfrak {M})}$, etc. It follows that all the operators induced by the sheaf morphisms above are local.

We turn our attention to the sheaf of microdifferential operators on $T^*\mathbb {R}^n$. For the definition and properties see [42, Section 17.5]. Following the arguments in [40, pp. 278-285] (cf.  also [42]) we see that sections of the sheaf $\Psi _a^{micro}$ of analytic microdifferential operators are acting on sections of $\textsf{D}^{micro}_{[\mathfrak {M}]}(\mathbb {R}^n)$ ($\mathfrak {M}$ is always a [semiregular] weight matrix) and they do so in a “local” manner: Let $\mathcal {U}$ be a conic subset of $T^*\mathbb {R}^n$, $\mathfrak {M}$ be a [semiregular] weight matrix, $[A]\in \Psi ^{micro}_a(\mathcal {U})$ and $[u]\in \mathfrak {D}^{micro}_{[\mathfrak {M}]}(\mathcal {U})$; then ${{\,\textrm{supp}\,}}[A][u]\subseteq [u]$ by Theorem 4.3. In fact Theorem 4.5 gives a more precise statement. We note that we can easily extend the notion of ${{\,\textrm{essupp}\,}}_{[\mathfrak {M}]}$ to analytic microdifferential operators [*A*]: $(x,\xi )\notin {{\,\textrm{essupp}\,}}_{[\mathfrak {M}]}[A]$ if there is a representative *A* of [*A*] defined in a conic neighborhood of (x,ξ) such that $(x,\xi )\notin {{\,\textrm{essupp}\,}}_{[\mathfrak {M}]}[A]$.

### Theorem 4.6

If $$[A]\in \Psi ^{micro}_a(\mathcal {U})$$ then

$$\begin{aligned} {{\,\textrm{supp}\,}}_{[\mathfrak {M}]} [A][u]\subseteq {{\,\textrm{supp}\,}}_{[\mathfrak {M}]} [u]\cap {{\,\textrm{essupp}\,}}_{[\mathfrak {M}]}[A]\qquad \;\,\forall \,[u]\in \mathfrak {D}^{micro}_{[\mathfrak {M}]}(\mathcal {U}) \end{aligned}$$

for all [semiregular] weight matrices $\mathfrak {M}$.

Similarly we can extend the notion of ellipticity to microdifferential operators. Since any elliptic analytic pseudodifferential operator has locally in the algebra of analytic pseudodifferential operators an inverse modulo analytic regularizing operators ([42, Proposition 17.2.21]) we obtain

### Theorem 4.7

Let $A\in \Psi _a^{micro}(\mathcal {U})$ be elliptic. Then

$$\begin{aligned} {{\,\textrm{supp}\,}}_{[\mathfrak {M}]} [A][u]={{\,\textrm{supp}\,}}_{[\mathfrak {M}]}[u]\qquad \;\,\forall \,[u]\in \mathfrak {D}^{micro}_{[\mathfrak {M}]}(\mathcal {U}) \end{aligned}$$

for every [semiregular] weight matrix $\mathfrak {M}$.

This proves Theorem 2.6. We finish this section by turning our attention to classic analytic pseudodifferential operators. For the definition and basic theorey we refer to [42, Chapter 17] and note here that the symbol *a* associated to a classical analytic pseudodifferential operator *A* of order *d* on $\Omega \times \Gamma$ is of the form

$$\begin{aligned} a(x,\xi )\sim a_d(x,\xi )+a_{d-1}(x,\xi )+a_{d-2}(x,\xi )+\cdots \end{aligned}$$

where the symbols $a_{d-j}(x,\xi )$ are homogeneous of degree d-j in the second variable. We call $a_d$ the principal symbol of *A* and can therefore define the characteristic set of *A*:Hence *A* is elliptic in the set $\mathcal {U}\setminus {{\,\textrm{Char}\,}}A$, cf. [42, Section 17.5]. We say that a section $[A]\in \Psi _a^{micro}(\mathcal {U})$ is a classical microdifferential operator on $\mathcal {U}$ if there is a representative *A* of [*A*] which is a classical pseudodifferential operator in $\mathcal {U}$. By [42, 17.5.2] the inverse of an elliptic classical microdifferential operator $[A]\in \Psi ^{micro}_a(\mathcal {U})$ is again a classical microdifferential operator. The following result is a direct consequence of Theorem 4.7.

### Theorem 4.8

Let *A* be a classical analytic pseudodifferential operator on some open set $\Omega \subseteq \mathbb {R}^n$ and $\mathfrak {M}$ be a [semiregular] weight matrix. Then

$$\begin{aligned} {{\,\textrm{WF}\,}}_{[\mathfrak {M}]} u={{\,\textrm{WF}\,}}_{[\mathfrak {M}]}Au\cup {{\,\textrm{Char}\,}}A\qquad \;\,\forall \,u\in \mathcal {E}^\prime (\Omega ). \end{aligned}$$

Note that all these considerations hold also if we replace $\mathbb {R}^n$ by a paracompact real-analytic manifold $\mathcal {M}$, cf. [42, Chapter 17].

## Hypoelliptic differential operators of principal type

In order to formulate the main results of the last two sections we need to recall some facts and notations from the modern geometric theory of linear partial differential equations (with real-analytic coefficients).

If $f\in \mathcal {C}^{\infty }(T^*\Omega \!\setminus \!\{0\})$ is a real-valued smooth function, then the Hamiltonian vector field associated to *f* is

$$\begin{aligned} H_f= \sum _{j=1}^n\left( \frac{\partial f}{\partial \xi _j}\frac{\partial }{\partial x_j} -\frac{\partial f}{\partial x_j}\frac{\partial }{\partial \xi _j}\right) . \end{aligned}$$

Now if *A* is a classical pseudodifferential operator in Ω with principal symbol $a_d=b+ic$ where *b* and *c* are real-valued, then a null bicharacteristic leaf of *A* is an integral manifold of the Lie algebra generated by *b* and *c*, which is contained in ${{\,\textrm{Char}\,}}A$. If the dimension of a null bicharacteristic leaf is 1 then we refer to it as a null bicharacteristic curve.

### Definition 5.1

([42, Definition 23.1.37]) We say that a differential operator *P* with principal symbol $p_d$ satisfies Condition $(\textbf{P})$ if ${{\,\textrm{Re}\,}}p_d$ is linear independent of the radial vector field on $T^*\Omega \setminus \{0\}$ on each point $x\in \{y\in T^*\Omega \setminus \{0\}{{\,\textrm{Re}\,}}p_d(y)=0\}$ and the restriction of ${{\,\textrm{Im}\,}}p_d$ to any null bicharacteristic curve of ${{\,\textrm{Re}\,}}p_d$ does not change sign.

We note here that a smooth hypoelliptic differential operator of principal type satisfies Condition $(\textbf{P})$ in $T^*\Omega \!\setminus \!\{0\}$, cf. [42, Proposition 24.2.2]. On the other hand Treves [38], see also [42, Theorem 24.2.1], proved that an analytic differential operator *P* of principal type is smooth hypoelliptic or analytic hypoelliptic if and only if the following condition is satisfied:

**Figure:**
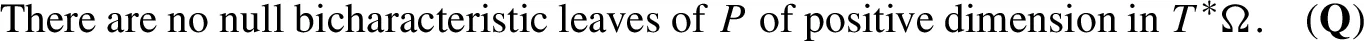

Thence Theorem 2.8 is a consequence of the following statement.

### Theorem 5.2

Let *P* be an analytic differential operator of principal type which satifies Condition $(\textbf{P})$. For any [semiregular] weight matrix $\mathfrak {M}$ the following statements are equivalent:

1. *P* satisfies Condition (**Q**).
2. *P* is $$[\mathfrak {M}]$$-hypoelliptic.

### Proof

Suppose (**Q**) holds and let $x_0\in \pi ({{\,\textrm{Char}\,}}P)$. By [42, (24.6.31)] we have in a suitably small neighborhood *U* of $x_0$

5.1 $$\begin{aligned} P(x,D_x)Au=Eu+Fu \end{aligned}$$

for all $u\in \mathcal {C}^\infty _0(U)$ where the distributional kernel of the operator $A:\mathcal {E}^\prime \rightarrow \mathcal {D}^\prime$ is analytic off the diagonal, *E* is an elliptic analytic pseudodifferential operator and *F* is analytic regularizing.

Let $U_0\Subset U$ and $\chi \in \mathcal {C}^\infty _0(U)$ with $\chi \vert _{U_0}\equiv 1$. Then

$$\begin{aligned} P(x,D)A \chi E^{-1}u=E \chi E^{-1} u +F_1u \end{aligned}$$

where $$F_1=F\chi E^{-1}$$. Thus

$$\begin{aligned} \left( P A \chi E^{-1} u\right) \vert _{U_0}= u+ F_2u \end{aligned}$$

for $u\in \mathcal {C}^\infty _0(U_0)$, cf. [42, Proposition 17.2.21]. Here $F_1,F_2$ are analytic regularizing in $U_0$, i.e. $F_j(\mathcal {E}^\prime (U_0))\vert _{U_0}\subseteq \mathcal {C}^\omega (U_0)$. We put

$$\begin{aligned} G=\left( A\chi E^{-1}\right) \vert _{U_0}. \end{aligned}$$

By transposition we obtain

$$\begin{aligned} G^{\top }P^{\top }u=u +F_3u \end{aligned}$$

for all $u\in \mathcal {E}^\prime (U_0)$. where $F_3$ is again analytic regularizing. Now note that the distribution kernels of $A^{\top }$ and $(E^{-1})^{\top }$ are also analytic off the diagonal. By Theorem 3.14 it therefore follows that $G^{\top }$ is $[\mathfrak {M}]$-pseudolocal:

$$\begin{aligned} {{\,\mathrm{sing\, supp}\,}}_{[\mathfrak {M}]} G^{\top }u\subseteq {{\,\mathrm{sing\, supp}\,}}_{[\mathfrak {M}]} u \end{aligned}$$

for all $u\in \mathcal {E}^\prime (U_0)$. This implies that ${{\,\mathrm{sing\, supp}\,}}_{[\mathfrak {M}]} P^{\top }u={{\,\mathrm{sing\, supp}\,}}_{[\mathfrak {M}]} u$ for all $u\in \mathcal {E}^\prime (U_0)$. Thence $P^{\top }$ is $[\mathfrak {M}]$-hypoelliptic at $x_0$, i.e.

$$\begin{aligned} x_0\notin {{\,\mathrm{sing\, supp}\,}}_{[\mathfrak {M}]}P^{\top }\;\Longrightarrow \; x_0\notin {{\,\mathrm{sing\, supp}\,}}_{[\mathfrak {M}]}u \end{aligned}$$

for all distributions *u* defined near $x_0$. Since (**Q**) is invariant under transposition we have proven the implication (1)⇒(2).

On the other hand suppose that (**Q**) is violated at some point $x_0$. If we argue in a small enough neighborhood *U* of $x_0$ then we can take the phase function φ and the formal solution $a(z,\lambda )=\sum _{j=0}^\infty e^{i\lambda \varphi (z)}\lambda ^{-j}a_j(z,\lambda )$ of $P(z,D_z)a(z,\lambda )$ from [42, Sections 24.3 & 24.5], see also [42, Chapter 23]. Here we extend the coefficients of *P* holomorphically to a complex neighborhood $\widetilde{U}$ of *U* in $\mathbb {C}^n$. In particular $\varphi \vert _{z_n=0}=z^\prime \xi _0^\prime + i|z^\prime |^2$. Moreover, for N>0, consider the approximate solution of the equation Pu=0 given by $e^{i\lambda \varphi }a^{[N]}$ where

$$\begin{aligned} a^{[N]}(z,\lambda )=\sum _{j\le \frac{\lambda }{N}}\lambda ^{-j}a_j(z,\lambda ). \end{aligned}$$

By [42, Lemma 24.5.1 & (24.5.7)] we have, for *U* small enough, that for any compact set K⊆U there is a constant $C_K>0$ such that

5.2 $$\begin{aligned} \;\,\forall \,\lambda \ge 1:\quad \sup _{x\in K} |e^{i\lambda \varphi } a^{[N]}(x,\lambda )|\le C_K \end{aligned}$$

for $N\ge C_K$. Moreover, [42, Proposition 24.5.2 & (24.5.)] indicates that for any large *N* there are an open neighborhood $U^\mathbb {C}_N\subseteq \mathbb {C}^n$ of the origin and $C_N>0$ such that

5.3 $$\begin{aligned} \lambda \ge 1:\quad \max _{K_1}\;|P(z,D_z)e^{i\lambda \varphi a^{[N]}}|\le C_N \end{aligned}$$

for any compact set $K_1\subseteq U^\mathbb {C}$.

We recall from [42, pp. 995–996] that $\partial _{x_j}\varphi (0)=0$ for j=1,⋯,n and by the initial conditions $\partial _{x_j}^2\varphi (0)\ne 0$ for j=1,⋯,n-1.

We observe for any $C^2$-functions *f*, *g* we have the following formula:

$$\begin{aligned} \begin{aligned} D_jD_k\bigl (e^{i\lambda f}g\bigr )&= -i\lambda \bigl (\partial _j\partial _k f\bigr )e^{i\lambda f}g + \lambda ^2\bigl (\partial _j f\bigr )(\partial _k f) e^{i\lambda f}g\\&\quad +\lambda (\partial _k f)e^{i\lambda f}D_jg +\lambda (\partial _j f)e^{i\lambda f}D_kg +e^{i\lambda }D_jD_kg. \end{aligned} \end{aligned}$$

We set now f=φ and $g=a^{[N]}$ for abritrary *N* and obtain that

$$\begin{aligned} D^\alpha \bigl (e^{i\lambda \varphi (0)}a^{[N]}(0)\bigr )=-i\lambda \partial ^\alpha \varphi (0) +D^\alpha a^{[N]}(0) \end{aligned}$$

for any $\alpha \in \mathbb {Z}_+^n$ with |α|=2. If now K⊆U is any compact set containing the origin we conclude that

5.4 $$\begin{aligned} \begin{aligned} \sum _{|\alpha |=2}\sup _{x\in K}|D^\alpha \bigl (e^{i\lambda \varphi (x)}a^{[N]}(x)\bigr )|&\ge \sum _{|\alpha |=2} |\lambda \partial ^\alpha \varphi (0)+D^\alpha a^{[N]}(0)|\\&\ge \sum _{|\alpha |=2}\left( \lambda |\partial ^\alpha \varphi (0)| -|D^\alpha a^{[N]}(0)|\right) \ge c_1\lambda -c_2 \end{aligned} \end{aligned}$$

for all $\lambda \ge 1$ and some constants $c_1,c_2>0$.

We fix now *N*, *U* and $U^\mathbb {C}$ such that both (5.2) and (5.3) hold. If we assume now that *P* is $[\mathfrak {M}]$-hypoelliptic in *U* than in particular the assumptions of Proposition 5.3 are satisfied and therefore inequality (5.5) below holds for Ω=U and $\widetilde{{\Omega }}=U^\mathbb {C}$. If we set $u_\lambda =e^{i\lambda \varphi a^{[N]}}$ then the right-hand side of (5.5) is bounded when $\lambda \rightarrow \infty$ for any choice of $K^\prime$ and $\widetilde{K}$. But by (5.4) we have that the left-hand side of (5.5) is unbounded when $\lambda \rightarrow \infty$ for any *K* containing the origin. Hence (5.5) is violated and therefore *P* cannot be $[\mathfrak {M}]$-hypoelliptic for any [semiregular] weight matrix $\mathfrak {M}$. □

### Proposition 5.3

Let *P* be a differential operator with analytic coefficients in a connected set $\Omega \subseteq \mathbb {R}^n$ and assume that all coefficients of *P* extends to the open set $\widetilde{\Omega }\subseteq \mathbb {C}^n$. We suppose that for any $u\in \mathcal {C}(\Omega )$ with $Pu\in \mathcal {C}^\omega (\Omega )$ we have that $u\in \mathcal {C}^2(\Omega )$. Then for all compact set $K\subseteq \Omega$ there are compact sets $K^\prime \subseteq \Omega$, $\widetilde{K}\subseteq \widetilde{{\Omega }}$ and a constant C>0 such that

5.5 $$\begin{aligned} \sum _{{|\alpha |}\le 2}\sup _{x\in K}|D^\alpha u(x)|\le C\left\{ \sup _{x\in K^\prime }|u(x)|+\sup _{z\in \widetilde{K}}|P(z,D)u(z)|\right\} \end{aligned}$$

for all $$u\in \mathcal {O}(\widetilde{\Omega })$$.

### Proof

Following [42, p. 37] we confer $\mathcal {C}^\omega (\Omega )$ with the following topology: Let $(\Omega _\nu )_{\nu }$ be a sequence of neighborhoods of Ω in $\mathbb {C}^n$ such that $\Omega _{\nu +1}\subseteq \Omega _{\nu }$ and $\bigcap _{\nu =1}^\infty \Omega _\nu =\Omega$. Moreover, we may assume that all $\Omega _\nu$ are connected Runge open sets and that $\Omega _1\subseteq \widetilde{\Omega }$. Obviously $\mathcal {O}(\Omega _\nu )\subseteq \mathcal {O}(\Omega _{\nu +1})$ and there is a canonical injection $\lambda _\nu :\mathcal {O}(\Omega _\nu )\rightarrow \mathcal {C}^\omega (\Omega )$ for each $\nu \in \mathbb {N}$. In fact,

$$\begin{aligned} \mathcal {C}^\omega (\Omega )=\bigcup _{\nu =1}^\infty \lambda _\nu \left( \mathcal {O}(\Omega _\nu )\right) \end{aligned}$$

as sets. We equip $\mathcal {C}^\omega (\Omega )$ with the inductive limit topology coming from the spectrum $(\mathcal {O}(\Omega _\nu ),\lambda _\nu )$. We may denote this topological space by $\mathcal {A}(\Omega )$. In particular, $\mathcal {A}(\Omega )$ is a webbed space by [26] and a seminorm *p* is continuous on $\mathcal {A}(\Omega )$ if and only if $p\circ \lambda _\nu$ is a continuous seminorm on $\mathcal {O}(\Omega _\nu )$ for all $\nu \in \mathbb {N}$ according to [15].

It follows instantly that the space $E=\mathcal {C}(\Omega )\times \mathcal {A}(\Omega )$ is also a webbed space, see [26]. We claim that the subspace  is webbed, too. For this it is enough to show that *F* is a sequentially closed subspace of *E* by [26]: Let $(u_j,f_j)\in F$ be a sequence which converges in *E*, i.e. $(u_j,f_j)\rightarrow (u,f)$, then $u_j\rightarrow u$ in $\mathcal {D}^\prime (\Omega )$, which immediately gives that $Pu_j\rightarrow Pu$ in $\mathcal {D}^\prime (\Omega )$. On the other hand we have also that $Pu_j=f_j\rightarrow f$ in $\mathcal {D}^\prime (\Omega )$ and therefore Pu=f in the sense of distributions, which means that (u,f)∈F.

By assumption the map $\lambda : F\rightarrow \mathcal {C}^2(\Omega )$ given by λ(u,f)=u is well-defined. Moreover, note that if $(u_j,f_j)\rightarrow 0$ in *F* and $\lambda (u_j,f_j)\rightarrow w$ in $\mathcal {C}^1(U)$ then w=0. Thus λ has closed graph. Now the closed-graph theorem in the form given in [26] applies and therefore λ is continuous. □

### Remark 5.4

We observe that the proof of Theorem 5.2 implies that if *P* is an analytic differential operator which satisfies (**P**) but not (**Q**) then near each point $x_0\in \Omega$ there is a continuous function *u* defined in a neighborhood *U* of $x_0$ such that *Pu* is real-analytic in *U*, but *u* is not $\mathcal {C}^2$ near $x_0$.

## Métivier operators

In this section we show how the pattern of proof in [30] can be modified to prove Theorem 2.9. Note that the proof on the operator side is the same as in the analytic category, only involving analytic pseudodifferential and analytic Fourier integral operators. We need only the that these operators do transform ultradifferentiable wavefronts sets in the correct way, which is true by Theorem 4.2 and Theorem 4.3, respectively.

In fact, will work in the setting of Okaji [31] which generalizes the setting of Métivier. We say that an analytic classical pseudodifferential operator *P* of order *d* in an open set $\Omega \subseteq \mathbb {R}^n$ with total symbol

$$\begin{aligned} p(x,\xi )=p_d(x,\xi +p_{d-1}(x,\xi )+\cdots \end{aligned}$$

defined in $\Omega \times \Gamma$, where $\Gamma \subseteq \mathbb {R}^n\!\setminus \!\{0\}$ is an open cone is an Métivier-Okaji Operator if the following conditions are satisfied:

1. There are two analytic conic manifolds $\Sigma _1,\Sigma _2\subseteq T^*\Omega \!\setminus \!\{0\}$ with the same codimension ν, which are regular involutive, $\Sigma =\Sigma _1\cap \Sigma _2$ is a conic, symplectic, analytic submanifold of $T^*\Omega \setminus \{0\}$ of codimension 2ν and for each $\rho \in \Sigma$ we have that $T_\rho \Sigma =T_\rho \Sigma _1\cap T_\rho \Sigma _2$.
2. For each point $\rho =(x_0,\xi _0)\in \Sigma$ there exists a conic neighborhood $\mathcal {U}\subseteq T^*\Omega \setminus \{0\}$ of ρ and $M,\mu \in \mathbb {N}$ such that
  $$\begin{aligned} \frac{|p_{d-j}(x,\xi )|}{{|\xi |}^{d-j}}&\le C\left( d_{\Sigma _1}(x,\xi )+\left( d_{\Sigma _2}(x,\xi )\right) ^{\mu }\right) ^{M-j(\mu +1)/\mu } \\ \frac{|p_d(x,\xi )|}{{|\xi |}^d}&\ge C^{-1}\left( d_{\Sigma _1}(x,\xi )+\left( d_{\Sigma _2}(x,\xi )\right) ^\mu \right) ^{M} \end{aligned}$$
  for  with *C* being a constant only depending on $\mathcal {U}$. Here $d_{\Sigma _j}(x,\xi )$, (j=1,2), denotes a distance function between $(x,\xi /{|\xi |})$ and $\Sigma _j\cap \{{|\xi |}=1\}$.
3. The operator *P* is hypoelliptic in Ω with loss of $M\mu /(\mu +1)$ derivatives.

Thus Theorem 2.9 is a special case of the following theorem.

### Theorem 6.1

Let *P* be a Metiver-Okaji operator in Ω. Then *P* is $[\mathfrak {M}]$-hypoelliptic for any [semiregular] weight matrix $\mathfrak {M}$.

If we follow the arguments in [31, pp. 490–491] then we see that, using an elliptic analytic Fourier integral operator *F*, we can assume that $x_0=0$, $\xi _0=e_n$, such that the transformed operator $\tilde{P}=FPF^{-1}$ has the form

6.1 $$\begin{aligned} \tilde{P}=\sum _j\sum _{({|\alpha |}/\mu )+{|\beta |}=M-j(\mu +1)/\mu }c_{\alpha \beta }{x^\prime }^\alpha D^\beta _{x^\prime } \end{aligned}$$

where $x^\prime =(x_1,\dotsc ,x_\nu ,0,\dotsc ,0)$ and $\xi _\nu =(\xi _1,\dotsc ,\xi _\nu ,0,\dotsc ,0)$, ($\nu \le n$) and the $c_\alpha \beta$ are classical analytic pseudodifferential operators of order $d+({|\alpha |}-{|\beta |}-\mu M)/(\mu +1)$. The sets $\Sigma _j$, j=1,2, are transformed to , .

Our assumptions on *P* lead to the following conditions:

6.2 $$\begin{aligned} |y^\prime |+|\eta ^\prime |\ne 0 \quad \Longrightarrow \quad \sum _{{|\alpha |}/\mu +{|\beta |}=M} c_{\alpha {|\beta |}}(x_0,\xi _0){y^\prime }^\alpha {\eta ^\prime }^\beta . \end{aligned}$$

For the next condition set $\sigma ^M_{x\xi }(P)=\sum _{j=0}^{M\mu /(\mu +1)} \sum _{{|\alpha |}/\mu +\beta =M-j(\mu +1)/\mu }c_{\alpha \beta }(x,\xi )(y^\prime )^{\alpha }D^\beta _{y^\prime }$.

6.3 $$\begin{aligned} \text {The kernel of } \sigma ^M_{x\xi }(P)\text { in } \mathcal {S}(\mathbb {R}^n) \text { is } \{0\}. \end{aligned}$$

Therefore in order to prove Theorem 6.1 it is enough to show the following statement.

### Theorem 6.2

Let *P* be of the form (6.1) in a conic neighborhood $\mathcal {U}$ of $(0,e_n)$. If *P* satisfies (6.2) and (6.3) then there is a conic neighborhood $\mathcal {V}$ of $(0,e_n)$ such that

$$\begin{aligned} {{\,\textrm{WF}\,}}_{[\mathfrak {M}]}u\cap \mathcal {V}={{\,\textrm{WF}\,}}_{[\mathfrak {M}]} u\cap \mathcal {V}\qquad \;\,\forall \,u\in \mathcal {E}^\prime (\Omega ) \end{aligned}$$

for any [semiregular] weight matrix $\mathfrak {M}$.

In order to prove Theorem 6.2 we need to continue to transform the operator *P*. We fix the following notation

$$\begin{aligned} A_j=\frac{\partial }{\partial x_j},\qquad A_{-j}=x_j\left( \frac{\partial }{\partial x_n}\right) ^{\tfrac{1}{\mu }},\qquad j=1,\dotsc ,\nu . \end{aligned}$$

For  we set $A_I=A_{j_1}\dotsc A_{j_k}$. Moreover we denote $|I_+|=\#\{j_k>0\}$, $|I_-|=\#\{j_k<0\}$ and $\langle I\rangle =|I_+|+(1/\mu )|I_-|$. Thus if *P* is of the form (6.1) then we can write

$$\begin{aligned} P(x,D_x)=\sum _{\langle I\rangle =M}c_{I}(x,D_x)A_I \end{aligned}$$

where $c_I$ are analytic pseudodifferential operators of order d-M in a neighborhood of $(x_0,\xi _0)$. If we multiply *P* with an elliptic factor and, if necessary, take powers of *P* then we can assume that d=M>ν.

As the next step we add new variables $x^{{\prime \prime }}=(x_{-\nu },\dotsc ,x_{-1})$ and and denote $\tilde{x}=(x^{{\prime \prime }},x)$ and $\tilde{\xi }=(\xi ^{\prime \prime },\xi )$. Let $\phi (x^{\prime \prime })\in \mathcal {C}^\infty _0(\mathbb {R}^\nu )$ be such that $\phi (x^{{\prime \prime }})=1$ near the origin. We extend a distribution $u\in \mathcal {D}^\prime (\mathbb {R}^n)$ to a distribution $\tilde{u}\in \mathcal {D}^\prime (\mathbb {R}^{n+\nu })$ by setting

$$\begin{aligned} \tilde{u}(\tilde{x})=\phi (x^{\prime \prime })u(x). \end{aligned}$$

The operators $A_j$ and $A_{-j}$ are extended by

$$\begin{aligned} \tilde{A}_j=\frac{\partial }{\partial x_j},\qquad \tilde{A}_{-j}=\left( \frac{\partial }{\partial x_{-j}}+x_j\frac{\partial }{\partial x_n}\right) \left( \frac{\partial }{\partial x_n}\right) ^{-(\mu -1)/\mu }. \end{aligned}$$

If we consider $c_I(x,\xi )$ as a symbol in some conic neighborhood of $(\tilde{x}_0,\tilde{\xi _0})$, $\tilde{x}_0=(0,x_0)$, $\tilde{\xi }_0=(0,\xi _0)$, which is independent of $(x^{\prime \prime },\xi ^{\prime \prime })$ then we can extend the operator *P* by

$$\begin{aligned} \tilde{P}(\tilde{x},D_{\tilde{x}})=\sum _{\langle I\rangle =M}\tilde{c}_I(\tilde{x},D_{\tilde{x}})\tilde{A}_I \end{aligned}$$

We observe that there are a neighborhood *U* of $x_0$ and a conic neighborhood $\tilde{\mathcal {U}}$ of $(\tilde{x},\tilde{\xi })$ such that for all $\tilde{u}\in \mathcal {E}^\prime (U)$ we have that

6.4 $$\begin{aligned} {{\,\textrm{WF}\,}}_{[\mathfrak {M}]} \widetilde{Pu}\cap \mathcal {U}={{\,\textrm{WF}\,}}_{[\mathfrak {M}]}\tilde{P}\tilde{u}\cap \mathcal {U}. \end{aligned}$$

Finally we consider the coordinate change $\tilde{x}\mapsto \tilde{y}=(y^{\prime \prime },y)$ given by

$$\begin{aligned} y^{\prime \prime }&=(y_{-\nu },\dotsc ,y_{-1})=(x_{-\nu },\dotsc ,x_{-1})\\ y&=(y_1,\dotsc ,y_{n-1},y_n)=\left( x_1,\dotsc ,x_{n-1},x_n-\frac{1}{2}\sum _{j=1}^\nu x_jx_{-j}\right) \end{aligned}$$

In the $\tilde{y}$-coordinates the operator $\tilde{P}$ transfroms to

$$\begin{aligned} Q\left( \tilde{y},D_{\tilde{y}}\right) =\sum _{\langle I\rangle =M}d_I(\tilde{y},D_{\tilde{y}}) X_I \end{aligned}$$

where the $d_I$ are operators of order 0 and

$$\begin{aligned} X_j&=\frac{\partial }{\partial y_j}-\frac{1}{2}y_{-j}\frac{\partial }{\partial y_{n}}\\ X_{-j}&=\left( \frac{\partial }{\partial _{-y_j}}+\frac{1}{2}y_j\frac{\partial }{\partial y_n}\right) \left( \frac{\partial }{\partial y_n}\right) ^{-(\mu -1)/(\mu )}. \end{aligned}$$

In this setting Okaji [31] proved the following theorem:

### Theorem 6.3

([31, Theorem 3]) Let N=n+ν and $\mathcal {U}\subseteq T^*\mathbb {R}^N\setminus \{0\}$ be a conic neighborhood of $(x_0,\xi _0)$, $x_0=0$ and $\xi _0=(0,\dotsc ,0,1)$. Assume that the operator *P* is defined in $\mathcal {U}$ and satisfies the following conditions:

- $P(x,D_x)=\sum _{\langle I\rangle =M}c_I(x,D_x)X_I$ where $c_I$ are classical pseudodifferential operators of order 0 in $\mathcal {U}$, $X_j=\frac{\partial }{\partial x_j}-\frac{1}{2}x_{j+\nu }\frac{\partial }{\partial x_N}$ and $X_{-j}=(\frac{\partial }{\partial x_{j+\nu }}+\frac{1}{2}x_j\frac{\partial }{\partial x_N})\left( \frac{\partial }{\partial x_N}\right) ^{-(\mu -1)/\mu }$ for $j=1,\dotsc ,\nu$ and $M\ge \nu + 1$.
- For any $\zeta \in \mathbb {R}^{2\nu }\setminus \{0\}$ we have $\sum _{\langle I\rangle =M}c_{I,0}(x_0,\xi _0)\zeta ^I\ne 0$ where $c_{I,0}$ denotes the principal symbol of the operator $c_I$.
- Setting $\textit{P}_{x\xi }(y,D_y)=\sum _{\langle I\rangle =M}\tilde{X}_I(y,D_y)$; $\tilde{X}_j=\frac{\partial }{\partial y_j}-\frac{1}{2}y_{j+\nu }$ and $\tilde{X}_{-j}=\frac{\partial }{\partial y_{j+\nu }}+\frac{1}{2}y_j$ then $\textit{P}_{x_0\xi _0}(y,D_y)$ is injective on $\mathcal {S}(\mathbb {R}^N)$.

Then there are a neighborhood *U* of $x_0$, a conic neighborhood $\mathcal {U}$ of $(x_0,\xi _0)$ and an operator $A\in {{\,\mathrm{a-Op}\,}}(S^{-M/\mu }_{1/(\mu +1),1/(\mu +1)})(U)$ such that for all $\varphi \in \mathcal {C}^\infty _0(U)$ and every $u\in \mathcal {E}^\prime (U)$ we have that

6.5 $$\begin{aligned} \mathcal {U}\cap {{\,\textrm{WF}\,}}_a(A\varphi u)=\emptyset . \end{aligned}$$

In Theorem 6.3${{\,\mathrm{a-Op}\,}}(S^{m}_{\rho ,\delta }(\Omega ))$ denotes a class of analytic pseudodifferential operators of type $(\rho ,\delta )$, $0<\rho \le 1$ and $0\le \delta <1$, introduced by Métivier [30]. A function $a\in \mathcal {C}^\infty (U\times U\times \mathbb {R}^N)$, $U\subseteq \mathbb {R}^N$ open, is in $a\in S^m_{\rho ,\delta }$ if there are C>0 and R>0 such that

$$\begin{aligned} |\partial ^{\alpha }_{x,y}\partial ^\beta _\xi a(x,y,\xi )|\le C^{{|\alpha |}+{|\beta |}+1} \left( 1+{|\xi |}\right) ^m\left( {|\alpha |}+{|\alpha |}^{1-\delta }{|\xi |}^\delta \right) ^{|\alpha |}\left( \frac{{|\beta |}}{{|\xi |}}\right) ^{\rho {|\beta |}} \end{aligned}$$

for all $\alpha \in \mathbb {Z}_+^{2N},\beta \in \mathbb {Z}_+^N$, x,y∈U and $\xi \in \mathbb {R}^N$ with $R{|\beta |}\le {|\xi |}$. Then the kernel of the associated operator $A={{\,\textrm{Op}\,}}a$ is

$$\begin{aligned} K_A=(2\pi )^{-N}\int e^{i(x-y)\xi }a(x,y,\xi )\,d\xi . \end{aligned}$$

According to [30, Lemma 3.3], cf. also [30, pp. 33-34] we have thatThence by Theorem 3.14

6.6 $$\begin{aligned} {{\,\textrm{WF}\,}}_{[\mathfrak {M}]}Au\subseteq {{\,\textrm{WF}\,}}_{[\mathfrak {M}]}u \end{aligned}$$

for all $u\in \mathcal {E}^\prime (U)$ and every [semiregular] weight matrix $\mathfrak {M}$. We conclude also from (6.5) that

6.7 $$\begin{aligned} \mathcal {U}\cap {{\,\textrm{WF}\,}}_{[\mathfrak {M}]} A\varphi u=\emptyset . \end{aligned}$$

Therefore combining (6.4), (6.6) and (6.7) proves Theorem 6.2.

## Data Availability

No datasets were generated or analysed during the current study.

## Appendix A. Differential operators with constant coefficients

### Theorem A.1

Let P=P(D) be an operator with constant coefficients in $\mathbb {R}^n$ and $\mathfrak {M}$ be a [semiregular] weight matrix. Then the following statements are equivalent:

1. The operator *P* is $[\mathfrak {M}]$-microhypoelliptic in any open set $\Omega \subseteq \mathbb {R}^n$, i.e. ${{\,\textrm{WF}\,}}_{[\mathfrak {M}]}Pu={{\,\textrm{WF}\,}}_{[\mathfrak {M}]}u$ for all $u\in \mathcal {D}^\prime (\Omega )$.
2. The operator *P* is $[\mathfrak {M}]$-hypoelliptic in any open set $\Omega \subseteq \mathbb {R}^n$.
3. For any open set $\Omega \subseteq \mathbb {R}^n$ we have that
4. Every fundamental solution *E* of *P* satisfies
  $$\begin{aligned} {{\,\mathrm{sing\, supp}\,}}_{[\mathfrak {M}]} E=\{0\}. \end{aligned}$$
5. There exists a fundamental solution *E* of *P* such that
  $$\begin{aligned} {{\,\mathrm{sing\, supp}\,}}_{[\mathfrak {M}]} E=\{0\}. \end{aligned}$$

### Proof

The implications $(1)\Rightarrow (2)\Rightarrow (3)\Rightarrow (4)\Rightarrow (5)$ are trivial. Suppose now that (5) holds and let $v\in \mathcal {E}^\prime (\mathbb {R}^n)$. Corollary 3.16 implies that

A.1 $$\begin{aligned} {{\,\textrm{WF}\,}}_{[\mathfrak {M}]} v= {{\,\textrm{WF}\,}}_{[\mathfrak {M}]} \bigl (E*(Pv)\bigr )={{\,\textrm{WF}\,}}_{[\mathfrak {M}]}Pv. \end{aligned}$$

Let $u\in \mathcal {D}^\prime (\Omega )$ be a distribution in some open set $\Omega \subseteq \mathbb {R}^n$. We continue by choosing an arbitrary point *p* and a neighborhood $U\Subset \Omega$. If $\chi \in \mathcal {C}^\infty _0(\Omega )$ is a test function with $\chi \vert _U=1$ then $(P(\chi u))\vert _U=(Pu)\vert _U$. Hence (A.1) implies for any $\xi \in \mathbb {R}^n\!\setminus \!\{0\}$ that

$$\begin{aligned} (p,\xi )\in {{\,\textrm{WF}\,}}_{[\mathfrak {M}]} u\Longleftrightarrow (p,\xi )\in {{\,\textrm{WF}\,}}_{[\mathfrak {M}]}Pu. \end{aligned}$$

It follows that *P* is $[\mathfrak {M}]$-microhypoelliptic in Ω. □

We will say that *P* is $[\mathfrak {M}]$-hypoelliptic if one of the equivalent conditions of Theorem A.1 is satisfied for some [semiregular] weight matrix $\mathfrak {M}$. It is easy to see that any $[\mathfrak {M}]$-hypoelliptic operator with constant coefficients is smooth hypoelliptic, cf. [41, Theorem 2.1]. More generally, the characterization in Theorem A.1 shows that $[\mathfrak {M}]$-hypoellipticity implies hypoellipticity for any larger ultradifferentiable class:

### Corollary A.2

Let *P* be a differential operator with constant coefficients.

- If *P* is $[\mathfrak {M}]$-hypoelliptic for some [semiregular] weight matrix then *P* is $[\mathfrak {N}]$-hypoelliptic for all [semiregular] weight matrices $\mathfrak {N}$ such that $\textbf{M}[\preceq ]\mathfrak {N}$.
- If *P* is $\{\mathfrak {M}\}$-hypoelliptic for some R-semiregular weight matrix then *P* is $(\mathfrak {N})$-hypoelliptic for any B-semiregular weight matrices $\mathfrak {N}$ with $\textbf{M}\{\lhd )\textbf{N}$.
- If *P* is $(\mathfrak {M})$-hypoelliptic for some B-semiregular weight matrix $\mathfrak {M}$ then *P* is $\{\mathfrak {N}\}$-hypoelliptic for all R-semiregular weight matrices $\mathfrak {N}$ such that $\mathfrak {M}\{\preceq \}\mathfrak {N}$.

The following Theorem is a slight generalization of [24, Theorem 11.4.7]. We may write $\textbf{M}\preceq \textbf{N}$ for two weight sequences if there is a constant C>0 such that $M_k\le C^{k+1}N_k$.

### Theorem A.3

Let $y\in \mathbb {R}^n$, *P* be a hypoelliptic differential operator with constant coefficients, $\mathfrak {M}$ be a countable collection of weight sequences, $\Omega \subseteq \mathbb {R}^n$ an open set and $x_0\in \Omega$. We assume that for any $u\in \mathcal {C}^\infty (\Omega )$ with Pu=0 there are some h>0 and $\textbf{M}\in \mathfrak {M}$ such that

$$\begin{aligned} |\langle y,D\rangle ^k u(x_0)|\le h^k M_k,\qquad k\in \mathbb {Z}_+^n. \end{aligned}$$

Then there are $\textbf{M}^0\in \mathfrak {M}$, s≥1 and C>0 such that $\textbf{G}^s\preceq \textbf{M}^0$ and

A.2 $$\begin{aligned} |\langle y,\zeta \rangle |\le C\bigl (1+|{{\,\textrm{Im}\,}}\zeta |\bigr )^s \end{aligned}$$

for all $\zeta \in \mathbb {C}^n$ satisfying P(ζ)=0.

### Proof

Let

Since *P* is hypoelliptic we have that $\mathcal {N}\subseteq \mathcal {C}^\infty (\Omega )$ and using [23, Theorem 4.4.2] we conclude that the topologies on $\mathcal {N}$ induced by $L^2_{loc}(\Omega )$ and by $\mathcal {C}^\infty (\Omega )$ coincide. For $r\in \mathbb {N}$ and $\textbf{M}\in \mathfrak {M}$ we set

which is a closed subset of the Frechet space $\mathcal {N}$. By assumption $\bigcup _{r\in \mathbb {N},\textbf{M}\in \mathfrak {M}} F_{r,\textbf{M}}=\mathcal {N}$. Baire’s Theorem implies that there have to be some $r_0\in \mathbb {N}$ and a weight sequence $\textbf{M}^0\in \mathfrak {M}$ such that $F_{r_0,\textbf{M}^0}$ includes an interior point. Since $F_{r_0,\textbf{M}^0}$ is convex and symmetric, the origin has to be an interior point. That means there have to be some δ>0 and a compact subset *K* of Ω such that $F_{r_0,\textbf{M}^0}$ includes all functions $u\in \mathcal {N}$ with $\Vert u\Vert _{L^2(K)}\le \delta$. Then

A.3 $$\begin{aligned} |\langle y,D\rangle ^k u(x_0)|\le r_0^{k} M^0_{k} \delta ^{-1}\Vert u\Vert _{L^2(K)},\qquad \;\,\forall \,u\in \mathcal {N}. \end{aligned}$$

Observe that (A.3) is homogeneous in *u* and obviously true for all $u\in \mathcal {N}$ with $\Vert u\Vert _{L^2(K)}=\delta$ and therefore for all $u\in \mathcal {N}$.

Now we can follow the end of the proof of [24, Theorem 11.4.7] verbatim to conclude that there is some s≥1 and C>0 such that

$$\begin{aligned} |\langle y,\zeta \rangle |\le C\left( 1+|{{\,\textrm{Im}\,}}\zeta |\right) ^s \end{aligned}$$

for all $\zeta \in \mathbb {C}^n$ with P(ζ)=0 and $\textbf{G}^s\preceq \textbf{M}^0$.

□

### Corollary A.4

Let *P* be a differential operator with constant coefficients and $\mathfrak {M}$ be a weight matrix. Then the following holds:

1. If $\textbf{M}$ is R-semiregular and *P* is $\{\mathfrak {M}\}$-hypoelliptic then there exists s≥1 such that *P* is $\{\textbf{G}^s\}$-hypoelliptic and $\{\textbf{G}^s\}\{\preceq \}\mathfrak {M}$.
2. If $\textbf{M}$ is B-semiregular and *P* is $(\mathfrak {M})$-hypoelliptic then there exists s≥1 such that *P* is $\{\textbf{G}^s\}$-hypoelliptic and $\{\textbf{G}^s\}\{\lhd )\mathfrak {M}$.

### Proof

If $\mathfrak {M}$ is *R*-semiregular and *P* is $\{\mathfrak {M}\}$-hypoelliptic then  by Theorem A.1. Hence we can apply Theorem A.3 and conclude for each j=1,⋯,n there are $\textbf{M}^j\in \mathfrak {M}$, $s_j\ge 1$ and $C_j$ such that

$$\begin{aligned} |\langle e_j,\zeta \rangle |\le C_j \left( 1+|{{\,\textrm{Im}\,}}\zeta |\right) ^{s_j} \end{aligned}$$

for all zeros ζ of the polynomial P(ζ) and $\textbf{G}^{s_j}\preceq \textbf{M}^{j}$.

Now let $s=\max s_j$ and $\textbf{M}^0$ the largest weight sequence of the collection .^2^ It follows first that $\textbf{G}^s\preceq \textbf{M}^0$ which by definition means that $\{\textbf{G}^s\}\{\preceq \}\mathfrak {M}$.

Moreover

$$\begin{aligned} |\zeta |\le \sum _{j=1}^n|\zeta _j|\le \sum _{j=1}^nC_j\left( 1+|{{\,\textrm{Im}\,}}\zeta |\right) ^{s_j}\le C\left( 1+|{{\,\textrm{Im}\,}}\zeta |\right) ^s \end{aligned}$$

where $C:=\sum C_j$. Hence [35, Theorem 2.2.2] implies that *P* is $\{\textbf{G}^s\}$-hypoelliptic.

In the Beurling case we need to return to the proof of Theorem A.3. By assumption *P* is $(\mathfrak {M})$-hypoelliptic and therefore

A.4

In particular, *P* is also hypoelliptic and therefore for each $j\in \{1,\dotsc ,n\}$ there is some $s_j\ge 1$ such that (A.2) holds for $y=e_j$ following the proof of [24, Theorem 11.4.7] (see also [35, Section 2.2]) As in the proof of the Roumieu case we conclude that *P* is $\{\textbf{G}^{s_0}\}$-hypoelliptic for $s_0=\max s_j$. Let $j_0$ be such that (A.2) holds for $y=e_{j_0}$ only for $s_0$. From (A.4) we infer that for all $u\in \mathcal {N}$, h>0 and every $\textbf{M}\in \mathfrak {M}$ there is some C>0 such that

$$\begin{aligned} |D_{j_0}^ku(x_0)|\le Ch^kM_k,\qquad k\in \mathbb {N}. \end{aligned}$$

Setting $u=e^{i\langle x,\zeta \rangle }$ for some ζ with P(ζ)=0 we obtain that for all h>0 and $\textbf{M}\in \mathfrak {M}$ there is some C>0 such that

A.5 $$\begin{aligned} |\zeta _{j_0}|^k\le Ch^k M_k,\qquad k\in \mathbb {Z}_+. \end{aligned}$$

As in the proof of [24, Theorem 11.4.7] we set  and recall that by the definition of $s_0$ the proof of [24, Theorem 11.4.7] gives that there is some b>0 such that $\mu (\tau )\ge b\tau ^{s_0}$ for large τ. Therefore (A.5) implies that

$$\begin{aligned} \tau ^{s_0 k}\le C\left( \frac{h}{b}\right) ^kM_k \end{aligned}$$

for large τ. Putting τ=k and using Stirling’s estimates we see that for each $\textbf{M}\in \mathfrak {M}$ and h>0 there is some C>0 such that

$$\begin{aligned} (k!)^{s_0}\le Ch^k M_k \end{aligned}$$

for large *k*. Thence $\textbf{G}^{s_0}\{\lhd )\mathfrak {M}$. □

If we consider the Gevrey matrix  then it follows directly that a differential operator with constant coefficients is hypoelliptic if and only if the operator is $\{\mathfrak {G}\}$-hypoelliptic. This assertion extends trivially to all classes which are larger than $\mathcal {E}^{\{ \mathfrak {G} \}} ( \Omega )$:

### Corollary A.5

Let *P* be a differential operator with constant coefficients and $\mathfrak {M}$ be a weight matrix. Then the following holds:

- If $\mathfrak {M}$ is R-semiregular and $\mathfrak {G}\{\preceq \}\mathfrak {M}$ then *P* is hypoelliptic if and only if *P* is $\{\mathfrak {M}\}$-hypoelliptic.
- If $\mathfrak {M}$ is B-semiregular and $\mathfrak {G}\{\lhd )\mathfrak {M}$ then *P* is hypoelliptic if and only if *P* is $(\mathfrak {M})$-hypoelliptic.

We conclude by considering the quasianalytic case. We recall that, if $\mathfrak {M}$ is a weight matrix then $\mathcal {E}^{\{ \mathfrak {M} \}} ( \Omega )$ is quasianalytic if and only if any $\textbf{M}\in \mathfrak {M}$ is a quasianalytic weight sequence, i.e. $\textbf{M}$ does not satifies ($\textbf{nq}$). In turn, $\mathcal {E}^{( \mathfrak {M} )} ( \Omega )$ is quasianalytic, if and only if there is some $\textbf{M}\in \mathfrak {M}$ which is quasianalytic.

If $\mathfrak {M}$ is an *R*-semiregular weight matrix with $\mathcal {E}^{\{ \mathfrak {M} \}} ( \Omega )$ being quasianalytic and *P* is $\{\mathfrak {M}\}$-hypoelliptic then necessarily s=1 in Theorem A.4(1), i.e. *P* is analytic hypoelliptic. On the other hand if $\mathfrak {M}$ is *B*-semiregular, $\mathcal {E}^{( \mathfrak {M} )} ( \Omega )$ is quasianalytic and *P* is $(\mathfrak {M})$-hypoelliptic then *P* is also analytic hypoelliptic by Corollary A.4(2). Since any differential operator *P* with constant coefficients is analytic hypoelliptic exactly when *P* is elliptic we conclude that the following statement holds, which generalizes the classical Theorem of Petrowsky [33] regarding analytic hypoellipticity.

### Corollary A.6

Let *P* be a differential operator with constant coefficients and $\mathfrak {M}$ be a [semiregular] weight matrix such that $\mathcal {E}^{[ \mathfrak {M} ]} ( \mathbb {R}^n )$ is quasianalytic. Then *P* is $[\mathfrak {M}]$-hypoelliptic if and only if *P* is elliptic.

## Appendix B. A semiregular quasianalytic class transversal to all semiregular Denjoy-Carleman classes

The following theorem is a generalization of the construction in [16, Subsection 7.4]. In this appendix $\mathcal {E}^{\{*\}}$, $*=\textbf{M},\mathfrak {M},\omega$, refers to the subsheaf of smooth functions generated by $\textbf{M}$, $\mathfrak {M}$ or ω (in the Roumieu sense).

### Theorem B.1

Let  be a ordered countable family of sequences $M^\lambda =(M_k^{(\lambda )})_{k\in \mathbb {Z}_+}$ of positive numbers such that

1. $$\sup _{k\in \mathbb {N}}\root k \of {M_k^{(\lambda )}}=+\infty $$ for all $$\lambda \in \mathbb {N}$$.
2. $M_k^{{(\lambda _1)}}\le M_k^{(\lambda _2)}$ for all $k\in \mathbb {Z}_+$ and all $\lambda _1\le \lambda _2$.

Then there exists a weight function ω in the sense of Braun-Meise-Taylor with the following properties:

- ω is semiregular (cf. Definition 2.3(2)).
- $$\mathcal {E}^{\{\omega \}}$$ is quasianalytic.
- $$\mathcal {E}^{\{\omega \}}\nsubseteq \mathcal {E}^{\{\mathfrak {M}\}}$$.

### Proof

We start by defining several auxiliary sequences: First we select two sequences $(a_\lambda )_{\lambda \in \mathbb {N}}$, $(b_\lambda )_{\lambda \in \mathbb {N}}$ iteratively. First we choose $a_1\in \mathbb {N}$ to be the smallest number such that $({M^{(1)}_{a_1}})^{2/a_1}\ge 1$ and set

$$\begin{aligned} \left( M_{a_1}^{(1)}\right) ^{2/a_1} \end{aligned}$$

which is possible by (1). Moreover, we iteratively select $a_k\in \mathbb {N}$ in such a way that

$$\begin{aligned} b_k:=\left( M_{a_k}^{(k)}\right) ^{2/a_k} \end{aligned}$$

satisfies the conditions $b_{k+1}\ge b_k$ and $b_k\ge k^2$ and

$$\begin{aligned} a_{k+1}\ge e^{b_k}a_k+1 \end{aligned}$$

for all $k\in \mathbb {N}$. This is again possible by (1). It follows that $k\mapsto a_k$ is strictly increasing and therefore $\lim a_k=+\infty$.

We set

$$\begin{aligned} q_k:=1, \quad 0\le k<a_1,\quad q_k:=b_{\lambda }^k,\quad a_\lambda \le k<a_{\lambda +1},\quad \lambda \in \mathbb {N}. \end{aligned}$$

We may set  and $\rho _k=q_k/q_{k-1}$.

**Claim:** The sequence $\rho _k$ is non-decreasing.In fact, if $k-1,k\in A_\lambda$ for some λ. then $\rho _k=b_\lambda$ and therefore $\rho _k$ is constant on the set . If $k-1\in A_{\lambda -1}$ and $k\in A_{\lambda }$ then $\rho _{k}=b^k_\lambda /b^{k-1}_{\lambda -1}$. Then $\rho _{k+1}=b_\lambda \ge \rho _k$ since $b_{\lambda -1}\ge b_\lambda$ and analogously $\rho _{k-1}= b_{\lambda -1}\le b_\lambda ^k/b_\lambda ^{k-1}$. Note that $\lim a_\lambda =\infty$.

Hence $\textbf{q}=(q_k)_k$ is a weight sequence and the same is true for $\textbf{Q}=(k!q_k)_k$. In fact $\lim _{k\rightarrow \infty } \root k \of {q_k}=\infty$, by construction, i.e. condition (2.4) holds for $\textbf{Q}$. Thus $\mathcal {C}^\omega (\Omega )\subseteq \mathcal {E}^{[ \textbf{Q} ]} ( \Omega )$.

**Claim:** $\mathcal {E}^{\{\textbf{Q}\}}\nsubseteq \mathcal {E}^{\{\mathfrak {M}\}}$. Let $\lambda \in \mathbb {N}$ then by definition we have for any $\nu \ge \lambda$ that

$$\begin{aligned} \left( \frac{Q_{a_\nu }}{M_{a_k}^{(\lambda )}}\right) ^{1/a_\nu } \ge \left( \frac{q_{a_\nu }}{M_{a_\nu }^{(\nu )}}\right) ^{1/a_\nu } =\frac{b_k}{\bigl (M_{a_\nu }^{a_\nu }\bigr )^{1/a_\nu }} =\left( M_{a_\nu }^{(\nu )}\right) ^{1/a_\nu }\ge \nu . \end{aligned}$$

It follows that

B.1 $$\begin{aligned} \sup _{k\in \mathbb {N}}\left( \frac{Q_k}{M_k^{\lambda }}\right) =\infty \end{aligned}$$

and therefore $\mathcal {E}^{\{\textbf{Q}\}}\nsubseteq \mathcal {E}^{\{\textbf{M}^{(\lambda )}\}}$ for any $\lambda \in \mathbb {N}$ by [34, Prop. 2.12(i)]. Using also [34, Prop. 4.6(i)] we see that $\mathcal {E}^{\{\textbf{Q}\}}\nsubseteq \mathcal {E}^{\{\mathfrak {M}\}}$. Note here that in order to apply these statements we need only that $\textbf{Q}$ is a weight sequence but not necessarily that any $\textbf{M}^{(\lambda )}$ satisfies (2.1).

**Claim:** The weight sequence $\textbf{Q}$ is quasianalytic.

Recall the asymptotic formula for the harmonic series $\sum _{k=1}^p=\log p+\gamma +\varepsilon _p$ where γ is the Euler constant and $\varepsilon _p\rightarrow 0$ if $p\rightarrow \infty$. Thus

$$\begin{aligned} \sum _{\ell \in A_\lambda }\frac{1}{\lambda } =\sum _{\ell =1}^{a_{\lambda +1}-1}\frac{1}{\ell } -\sum _{\ell =1}^{a_\lambda -1}\frac{1}{\ell } =\log (a_{\lambda +1}-1)-\log (a_\lambda -1)+\varepsilon _{a_{\lambda +1}-1}-\varepsilon _{a_\lambda -1}, \end{aligned}$$

and therefore the following estimate holds for any λ by construction:

$$\begin{aligned} \sum _{\ell \in A_\lambda }\frac{1}{(Q_\ell )^{1/\ell }} \ge \sum _{\ell \in A_\lambda }\frac{\ell }{(q_\ell )^{1/\ell }} =\frac{1}{b_\lambda }\sum _{\ell \in A_\lambda }\frac{1}{\ell }. \end{aligned}$$

On the other hand by the choice of $a_{\lambda +1}$ we see that

$$\begin{aligned} \frac{1}{b_\lambda }\log \left( \frac{a_{\lambda +1}-1}{a_\lambda -1}\right) \ge 1 \end{aligned}$$

for any $\lambda \ge 2$. Thence  and thus $\textbf{Q}$ is quasianalytic, because ($\textbf{nq}^\prime$) holds exactly when the series in ($\textbf{nq}$) is divergent, cf. e.g. [23] or [36, Theorem Theorem 19.11].

If we consider now the weight function $\omega _{\textbf{Q}}$ associated to the weight sequence $\textbf{Q}$, given by (4.7), then it is well known that $\omega _{\textbf{Q}}$ satisfies all conditions in Definition 2.2 except ($\omega _1$), cf. [37, Lemma 2.3] and the literature cited there. However, since the sequence $\textbf{Q}$ is strongly log-convex, i.e. the sequence $\textbf{q}$ satisfies (2.1), the associated weight function $\omega _{\textbf{Q}}$ is subadditive by [37, Theorem 4.5]. This implies that $\omega _{\textbf{Q}}$ satisfies ($\omega _1$), i.e. $\omega _{\textbf{Q}}$ is a weight function in the sense of Braun-Meise-Taylor. Moreover, $\omega _{\textbf{Q}}$ is semiregular: According to [37, Lemma 2.3] the property $\lim _{k\rightarrow \infty } (q_k)^{1/k}=\infty$ implies that $\omega _{\textbf{Q}}(t)=o(t)$ for $t\rightarrow \infty$.

Since $\mathcal {E}^{\{\textbf{Q}\}}\subseteq \mathcal {E}^{\{\omega _{\textbf{Q}}\}}$, cf. [37], it follows that $\mathcal {E}^{\{\omega _{\textbf{Q}}\}}\nsubseteq \mathcal {E}^{\{\mathfrak {M}\}}$. Finally, the quasianalyticity of $\textbf{Q}$ implies that

$$\begin{aligned} \int _{1}^{\infty }\frac{\omega _{\textbf{Q}}(t)}{1+t^2}\,dt=\infty , \end{aligned}$$

see [25, Lemma 4.1], which characterizes the quasianalyticity of $\mathcal {E}^{\{\omega _{\textbf{Q}}\}}$, cf. [34, Section 5]. □

### Corollary B.2

There exists a weight function ω in the sense of Braun-Meise-Taylor such that $\mathcal {E}^{\{\omega \}}$ is quasianalytic and

$$\begin{aligned} \mathcal {E}^{\{\omega \}}\nsubseteq \mathcal {E}^{\{\textbf{M}\}} \end{aligned}$$

for any semiregular weight sequence $\textbf{M}$.

### Proof

If $\textbf{M}$ is a semiregular weight sequence then (2.5) gives that there is a constant ρ>0 such that $M_k\le \rho ^{k^2+k}$. Thence $\textbf{M}\preceq \textbf{N}^\rho$, where $\textbf{N}^\rho$ is the weight sequence given by $N^\rho _k=\rho ^{k^2}$. If  then this implies that $\mathcal {E}^{\{\textbf{M}\}}\subseteq \mathcal {E}^{\{\mathfrak {N}\}}$ for any semiregular weight sequence $\textbf{M}$. However, it is easy to see that $\mathcal {E}^{\{\mathfrak {N}\}}=\mathcal {E}^{\{\widehat{\mathfrak {N}}\}}$ where  and that $\mathfrak {M}=\widehat{\mathfrak {N}}$ satisfies the conditions in Theorem B.1. By Theorem B.1 there exists therefore a semiregular weight function ω such that $\mathcal {E}^{\{\omega \}}$ is quasianalytic and $\mathcal {E}^{\{\omega \}}\nsubseteq \mathcal {E}^{\{\mathfrak {N}\}}$. □

### Remark B.3

It is worth pointing out that by construction the weight function ω in Theorem B.1 resp. Corollary B.2 is not only semiregular but also subadditive. For example, that means that $\mathcal {E}^{\{\omega \}}$ can be characterized by almost-analytic extensions according to [16, Theorem 1.5] and that it satisfies the assumptions of [1, Theorem 4.8] and [16, Theorem 7.7], cf. [16, Theorem 4.8].

## Footnotes

1 We use here the classical definition of *principal type* as used in [38], which differs from the modern definition, e.g. [42, Definition 23.1.2].
